# Differential prognostic impact of interleukin-34 mRNA expression and infiltrating immune cell composition in intrinsic breast cancer subtypes

**DOI:** 10.18632/oncotarget.25226

**Published:** 2018-05-01

**Authors:** Karin Zins, Gerwin Heller, Mathias Mayerhofer, Martin Schreiber, Dietmar Abraham

**Affiliations:** ^1^ Division of Cell and Developmental Biology, Center for Anatomy and Cell Biology, Medical University of Vienna, A-1090 Vienna, Austria; ^2^ Department of Medicine I, Clinical Division of Oncology, Medical University of Vienna, A-1090 Vienna, Austria; ^3^ Department of Obstetrics and Gynecology, Medical University of Vienna, A-1090 Vienna, Austria; ^4^ Comprehensive Cancer Center Vienna, A-1090 Vienna, Austria

**Keywords:** IL-34, CSF-1, gene expression, breast cancer patients, PAM50 subclasses

## Abstract

Interleukin-34 (IL-34) is a ligand for the CSF-1R and has also two additional receptors, PTPRZ1 and syndecan-1. IL-34 plays a role in innate immunity, inflammation, and cancer. However, the role of IL-34 in breast cancer is still ill-defined. We analyzed IL-34 mRNA expression in breast cancer cell lines and breast cancer patients and applied established computational approaches (CIBERSORT, ESTIMATE, TIMER, TCIA), to analyze gene expression data from The Cancer Genome Atlas (TCGA). Expression of IL-34 was associated with a favorable prognosis in luminal and HER2 but not basal breast cancer patients. Gene expression of CSF-1 and CSF-1R was strongly associated with myeloid cell infiltration, while we found no or only weak correlations between IL-34, PTPRZ1, syndecan-1 and myeloid cells. *In vitro* experiments showed that tyrosine phosphorylation of CSF-1R, ERK, and FAK and cell migration are differentially regulated by IL-34 and CSF-1 in breast cancer cell lines. Collectively, our data suggest that correlation of IL-34 gene expression with survival is dependent on the molecular breast cancer subtype. Furthermore, IL-34 is not associated with myeloid cell infiltration and directly regulates breast cancer cell migration and signaling.

## INTRODUCTION

Breast cancer is the most common cancer among women worldwide and remains the leading cause of cancer death among females [1]. Its malignancy grade and patient prognosis is not only influenced by various mutations that occur in the tumor cell, but also by the tumor microenvironment (TME) [2].

The TME of breast cancer consists of a heterogeneous collection of cells and is enriched in highly active immune cells which, together with cytokines, play an important role in the regulation of breast cancer [3, 4]. The host immune response during breast cancer is dynamic and can affect tumor growth both in promotive and suppressive ways. Consequently, the prognosis of breast cancer patients is influenced by the density, composition and activity of the tumor immune infiltrate [3]. The addition of tumor-promoting inflammation as the seventh hallmark of cancer reflects the double-edged role of inflammatory processes in cancer progression [5].

Macrophages, the most prevalent immune cells in mammary tumors, exert a profound influence at each stage of cancer progression [4]. The majority of macrophages is regulated by colony-stimulating factor-1 (CSF-1), a key growth factor modulating macrophage proliferation, survival, and function through interaction with its receptor CSF-1R (CD115) [6]. CSF-1R is the product of the receptor tyrosine kinase *c-fms* proto-oncogene [7]. Our previous breast cancer studies found that CSF-1/CSF-1R signaling promotes tumor growth [8] [9, 10] and it has been demonstrated that CSF-1R blockade using antibodies reduced the number of resident tumor-associated macrophages (TAMs) in tumors [11].

The discovery in 2008 of IL-34 as a new ligand of CSF-1R [12] has changed the existing functional biological concepts for CSF-1/CSF-R1 [13]. Like CSF-1, IL-34 promotes the survival and proliferation of monocytes, as well as their differentiation into macrophages [12] and both cytokines can polarize macrophages into immunosuppressive M2 macrophages [14]. In addition, IL-34 has been shown to be involved in areas as diverse as neuronal protection, autoimmune diseases, infection, cancer, degenerative bone diseases and immune tolerance [15].

Several studies have shown a correlation between high IL-34 expression level and tumor development [15]. A study in giant cell tumors of bone has revealed that the pathogenesis results directly from the supporting action of IL-34 on osteoclastogenesis [16]. In osteosarcoma, IL-34 has been shown to be rather involved in TAM recruitment [17]. IL-34 produced by cancer cells, has also been identified as a driver of chemoresistance [18]. Cytotoxic therapies have been shown to induce the production of IL-34 in breast cancer [19]. In hepatocellular carcinoma patients, high IL-34 levels have been associated with a poor prognosis, with shorter overall survival (OS) and time to recurrence [20].

However, IL-34 signaling cannot be considered as a simple equivalent of CSF-1/CSF-1R signaling. Recent studies have demonstrated that IL-34 also binds to other receptors, the receptor-type protein-tyrosine phosphatase zeta (PTPRZ1)[21] and syndecan-1 (CD138) [22], increasing the complexity. These findings suggest that IL-34 may also exert specific functions independently of the CSF-1R. Activation of the cell surface chondroitin sulfate (CS) proteoglycan PTPRZ1 leads to increased tyrosine phosphorylation of several signaling pathways and is upregulated in many human cancers, such as lung cancer, prostate cancer, and glioma, regulating cancer cell migration and metastasis [23–25]. IL-34 binding to syndecan-1 modulates the IL-34-induced CSF-1R signaling pathways, and IL-34 induces the migration of monocytes and macrophages in a syndecan-1-dependent manner [22]. Syndecan-1 is a cell surface heparin sulfate proteoglycan, which is expressed by many cancers [26]. In breast cancer, increased cell-membrane syndecan-1 levels are found [27] and it is associated with high-grade tumors [28].

Despite the known expression of CSF-1 and CSF-1R in human breast cancer and their clear therapeutic potential, the role of IL-34 remains unclear. Here, we measured the levels of IL-34 in breast cancer patients using qRT-PCR and assessed the association of IL-34 expression with breast cancer outcome. To explore their potential biological role, we studied the association between IL-34, CSF-1 and their receptors with immune cell infiltration based on the breast cancer dataset of The Cancer Genome Atlas (TCGA). We report that IL-34 expression is associated with differential outcome in intrinsic breast cancer subtypes. Our *in vitro* experiments provide evidence that IL-34 regulates cancer cell migration and mediates signaling in human breast cancer cells.

## RESULTS

### IL-34 gene expression in normal and tumor tissue

**Figure 1:**
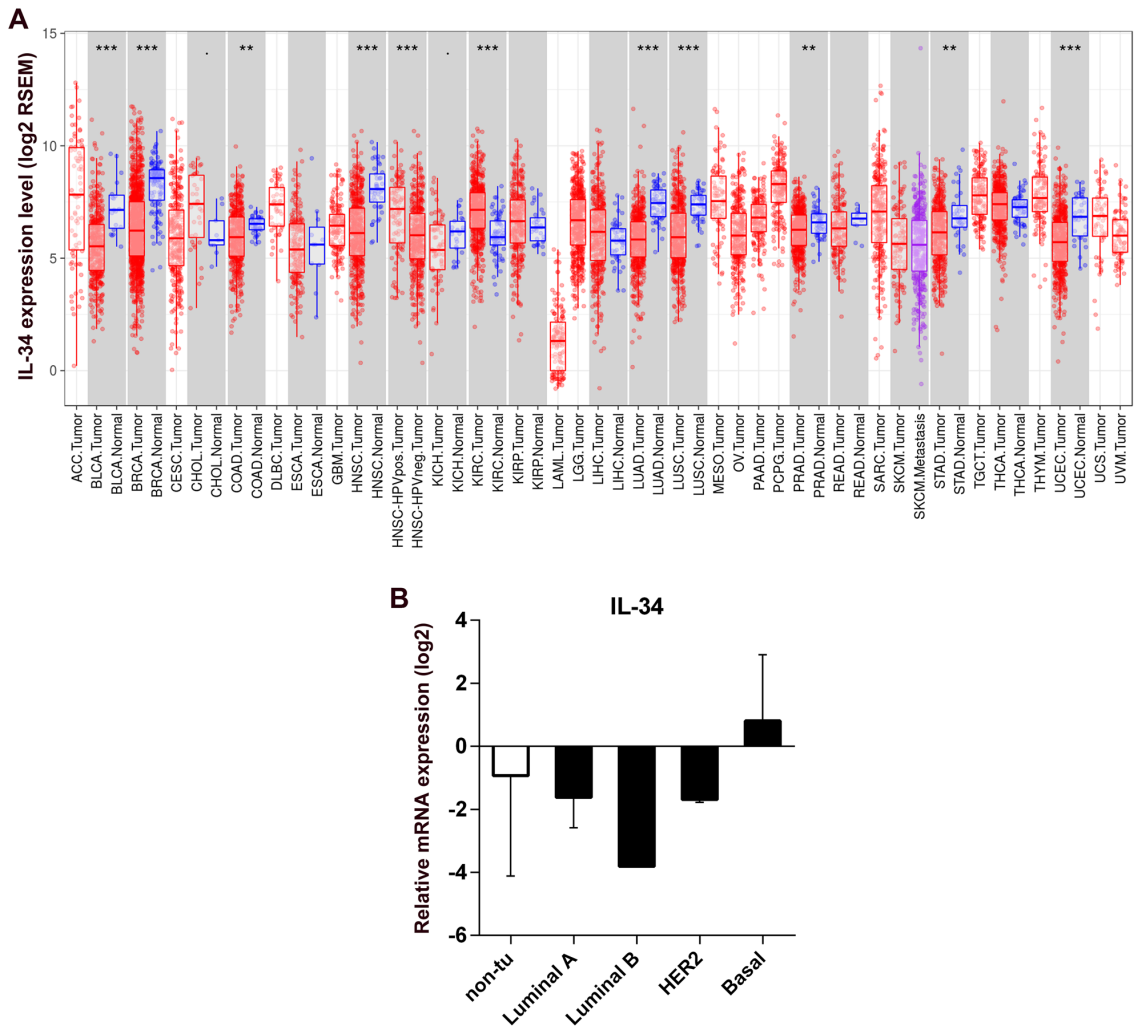
IL-34 mRNA expression in normal tissue, cancerous tissue, and breast cancer cell lines **(A)** RNA expression overview shows RNA-seq data from The Cancer Genome Atlas (TCGA). Datasets of normal and cancerous human tissues were obtained from the TCGA database. Boxplots show the distributions (median, spread and outliers) of the IL-34 mRNA levels (log2) by the RNAseq by Expectation-Maximization (RSEM) normalization across normal and cancerous tissue. **(B)** IL-34 mRNA expression across molecular subtypes of breast cancer cell lines and normal breast cell lines. IL-34 expression level reported as log2 values +/− SD according to the molecular subtype of cell lines; non-tu, non-tumorigenic cell lines.

We analyzed differential IL-34 gene expression of RNA-seq data from normal tissues and tumor tissues using data generated by The Cancer Genome Atlas (TCGA). Summary of the distributions of the gene expression values were presented by boxplots in Figure 1A with the median, spread and outliers showing for each gene. IL-34 expression was distinctly separated between the normal and tumor tissues. In normal tissue, highest median IL-34 levels were found in normal breast tissue. In breast cancer tumors abundant IL-34 expression variations were observed indicating that different gene expression patterns may exist in breast cancer tissues.

### IL-34 is differentially expressed in human breast cancer cell lines

These data, however, could not address the question of whether cancer cells contribute to expression of IL-34. To analyze the expression of IL-34 by cancer cells, we examined IL-34 expression in 14 breast cancer cell lines and 4 normal breast epithelial cell lines. Quantitative qRT-PCR showed detectable levels of IL-34 mRNA in different cancer cells with some variations. Basal-like cell lines (n = 6) showed higher IL-34 expression levels than HER2-type cell lines (n = 3), luminal cell lines (n = 5), and normal cell lines (n = 4). IL-34 expression was similar between luminal and HER2 cell lines, but showed IL-34 downregulation when compared to normal breast cancer cell lines (Table 1, Figure 1B). These observations suggest that IL-34 expression in breast cancer cells may differ depending on their molecular subtype.

**Table 1:** IL-34 mRNA expression in a panel of human breast cancer cell lines

Cell line	Cell type	ER^*^	PR^*^	HER2^*^	Vimentin^*^	N-cad^*^	IL-34 log2	Subtype^*^
HMEC	normal						3,16	
Hs 578Bst	normal						−3,80	
MCF 10A	F						−3,08	
MCF 10F	F						0,00	
MCF7	tumor	+	+	−	−	−	−2,62	Luminal A
ZR-75-1	tumor	+	+	−	−		−0,82	Luminal A
T-47D	tumor	+	+	−	−		−2,28	Luminal A
CAMA-1	tumor	+	+	−	−		−0,76	Luminal A
BT-474	tumor	+	+	+	−		−3,81	Luminal B
SK-BR-3	tumor	−	−	+	−	−	−1,62	HER2
AU565	tumor	−	−	+			−1,75	HER2
MDA-MB-453	tumor	−	−	+	−	−	n.a.	HER2
MDA-MB-231	tumor	−	−	−	+	−	−0,76	Basal
MDA-MB-468	tumor	−	−	−	−		1,47	Basal
CAL-51	tumor	−		−			−1,78	Basal
HCC1143	tumor			−			3,74	Basal
HCC1937	tumor	−	−	−			2,42	Basal
Hs 578T	tumor	−	−	−	+	+	−0,22	Basal

F, fibrocystic disease; ER, estrogen receptor; PR, progesterone receptor; HER2, human epidermal growth factor receptor-2; N-cad, N-cadherin IL-34, interleukin-34; normal, untransformed human mammary epithelial cells; n.a., not applicable.

^*^Determined from references [65–70].

### Association of IL-34 expression with clinical and histopathological characteristics of breast cancer patients

**Figure 2:**
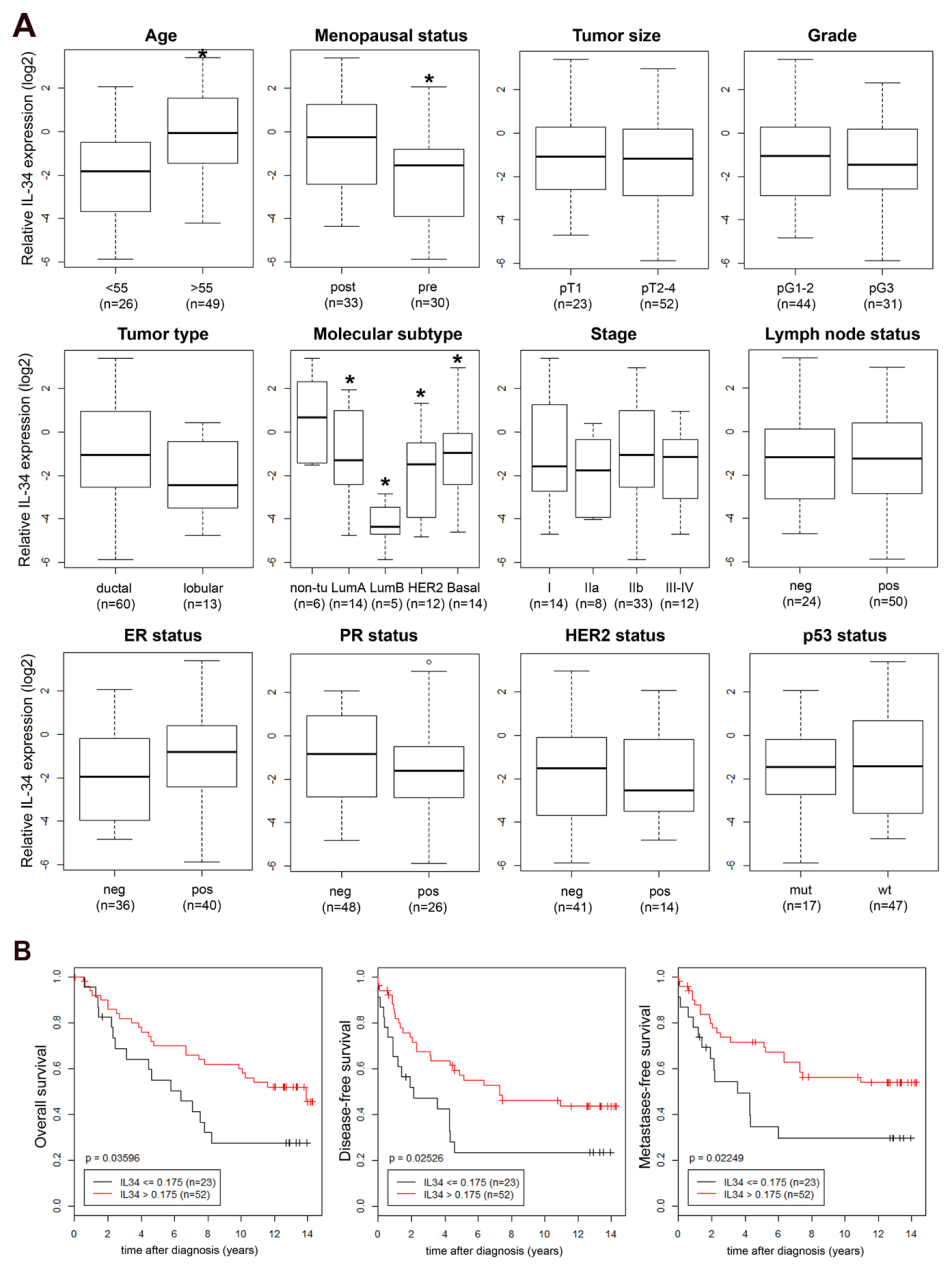
**(A)** IL-34 mRNA expression in breast tumors. Association of relative IL-34 mRNA expression (log2) with established clinical and histopathological parameters was analyzed in breast tumors. Boxplots of IL-34 expression in patients with an age at breast cancer onset of <55 vs. ≥55 years (^*^, *p*  =  0.002), in pre- vs. post-menopausal patients (^*^, *p*  =  0.007), in pT1 vs. pT2–4 breast tumors, in grade 1-2 vs. 3 breast tumors, IL-34 expression in ductal vs. lobular tumor type, in normal breast tissue (non-tumorous; non-tu) vs indicated molecular breast cancer subtypes (^*^, *p* = 0.005, ANOVA), in patients with the indicated tumor stages, in breast tumors from patients with a negative (neg, pN0) vs. a positive (pos, pN+) lymph node status, in estrogen receptor (ER) neg vs. pos tumors, in progesterone receptor (PR) neg vs. pos tumors, in HER2 neg vs. pos tumors, and in p53 mutant (mut) vs. wildtype (wt) tumors. Numbers in parentheses indicate the number of patients in each group. All *p*-values were determined via unpaired, two-sided *t*-tests except in molecular subtype and stage (ANOVA); neg, negative; pos, positive. Molecular subtype based on expression of the PAM50 gene set [71], determined with Affymetrix U133 Plus 2.0 GeneChips. **(B)** Association of IL-34 expression with survival of breast cancer patients. Kaplan–Meier analyses of the overall (left), disease-free (center) and metastasis-free survival (right) in breast cancer patients (n = 75) are shown. Patients were stratified into two groups according to IL-34 high and IL-34 low expression levels.

Next, we asked whether IL-34 expression could be detected in primary human breast cancers. We quantified relative IL-34 mRNA expression in primary tumor tissue samples of 75 patients by qRT-PCR and searched for correlations between IL-34 mRNA expression and clinical and histopathological characteristics of breast cancer patients. As shown in Figure 2A, significantly elevated mean IL-34 mRNA levels were associated with an age ≥55 years (*p* = 0.002) and post-menopause (*p* = 0.007). No correlation was found with tumor size, pathological type, tumor stage, tumor grade, lymph node status, estrogen receptor (ER) status, progesterone receptor (PR) status, HER2 status, and p53 status. Regarding the molecular subtypes, we observed the lowest IL-34 levels in luminal B cases. IL-34 levels in luminal A, HER-2 and basal-like cases were lower as compared to normal-like cases (*p* = 0.005, ANOVA), a similar pattern to the results of our analysis of breast cancer cell lines (Table 1 and Figure 1B; see above). Based on the mean Ct (cycle threshold) as a relative measure of the concentration of target in the PCR reaction, IL-34 gene expression was lower in cancer cell lines (mean Ct 30.94) as compared to our tumor samples (mean Ct 26.35) and normal tissue (mean Ct 26.26).

These data suggest that IL-34 expression varies significantly among different molecular subtypes of breast cancer and that cancer cells only partially contribute to IL-34 gene expression levels.

### Association of IL-34 expression with breast cancer prognosis

Detailed follow-up records were available for our 75 patients. We subjected these patients to Kaplan–Meier analyses of the overall (OS), disease-free (DFS), and metastasis-free survival (MFS), comparing IL-34-high patients with IL-34-low patients (Figure 2B). Unexpectedly, IL-34-high patients exhibited a better prognosis. Patients with a high IL-34 expression showed a significantly better OS (*p* = 0.036) than IL-34-low patients (Figure 2B). Likewise, high IL-34 expression had a significant impact on DFS and MFS as well. High IL-34 expression was associated with a significantly better prognosis in both, the analyses of the DFS (*p* = 0.025) and the MFS (*p* = 0.022) (Figure 2B). In conclusion, patients with a high relative IL-34 mRNA expression exhibited increased survival and better prognosis.

### Association of IL-34 expression with breast cancer prognosis is dependent on the molecular subtype

**Figure 3:**
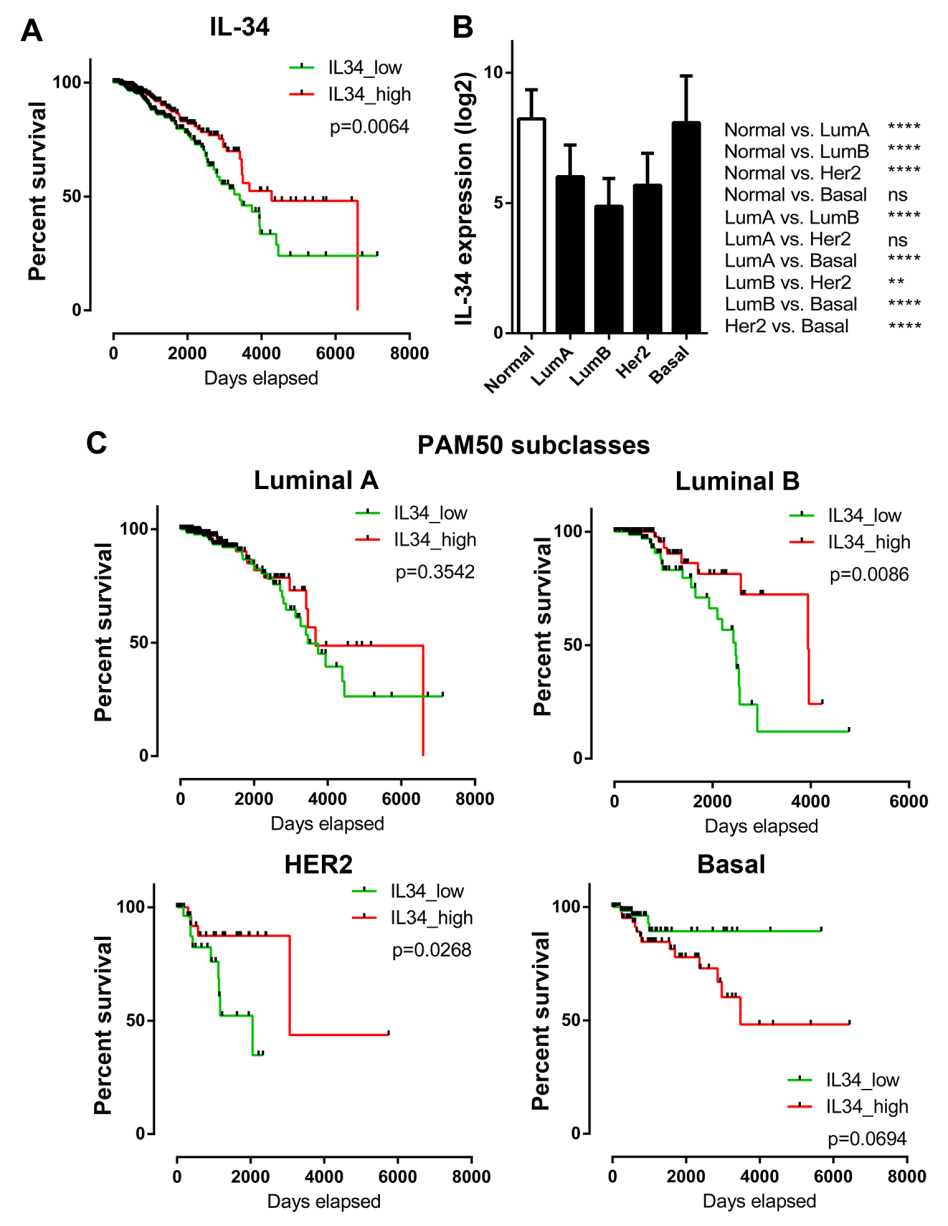
IL-34 expression and overall survival across molecular subtypes of breast cancer **(A)** Kaplan–Meier plots of overall survival in IL-34-high and IL-34-low expressing TCGA BRCA patients (n = 1056). Median IL-34 expression was used as cut-off for group separation. Log rank tests were calculated. **(B)** IL-34 mRNA expression in normal breast tissue and in PAM50 breast cancer subclasses. The bar graph shows the IL-34 mRNA levels (log2) across normal breast tissue (n = 113) and the molecular subtypes of breast cancer (luminal A, n = 412; luminal B, n = 188; HER2-enriched, n = 64; basal, n = 140) of the TCGA BRCA dataset. Kruskal–Wallis and Dunn’s multiple comparison tests were calculated. Error bars indicate standard deviations. ^*^, *p* < 0.05; ^**^, *p* < 0.01; ^***^, *p* < 0.001; ^****^, *p* < 0.0001. **(C)** Kaplan–Meier plots of overall survival in IL-34-high and IL-34-low expressing TCGA BRCA patients stratified by PAM50 subclasses (luminal A, n = 412; luminal B, n = 188; HER2-enriched, n = 64; basal, n = 140). Median IL-34 expression was used as cut-off for group separation. Log rank tests were calculated.

To further elucidate the relationship between IL-34 and breast cancer, we used breast cancer data generated by The Cancer Genome Atlas (TCGA). The median IL-34 expression was correlated with OS of patients (median follow-up time: 671 days). Kaplan–Meier survival curves generated for IL-34 were split into high (top 50%) and low (bottom 50%) IL-34 expression, as shown in Figure 3A. These curves showed that high IL-34 expression was correlated with better outcome (log-rank *p =* 0.0064) confirming data from our own patient data set.

We then investigated whether IL-34 expression differs between distinct molecular breast cancer subtypes and if IL-34 expression is associated with OS in these subtypes. Analysis of TCGA breast cancer RNA-seq data sets showed that IL-34 expression was lowest in luminal B subtype (*p* < 0.0001 vs. normal). IL-34 levels in luminal A and HER-2 subtypes were also lower as compared to normal breast tissue (*p* < 0.0001 vs. normal). In contrast, basal-like cases showed the highest IL-34 expression amongst all subtypes (*p* < 0.0001 vs. other subtypes; ns. vs. normal) (Figure 3B). These data are in accordance with findings of our own patient dataset, showing that IL-34 gene expression differs strongly between breast cancer subtypes.

Kaplan–Meier OS analyses of patients split by molecular subtype and classified as belonging to IL-34 high or low showed significant survival differences (Figure 3C). Patients belonging to the luminal A subtype did not reveal a significant association between IL-34 expression and survival. Luminal B subtype patients showed a significantly better survival when IL-34 was high (log-rank *p =* 0.0086). Similarly, in the HER2 subtype high IL-34 expressing patients had a longer OS (log-rank *p =* 0.0268). In contrast, the basal subgroup revealed an association between IL-34-high expression and poor survival of breast cancer patients, however, this difference did not reach statistical significance (log-rank *p =* 0.0694). Thus, IL-34 mRNA expression levels are associated with differential prognosis in PAM50 breast cancer subtypes in the TCGA dataset.

### Association with overall survival: Expression of CSF-1 and IL-34-receptors

**Figure 4:**
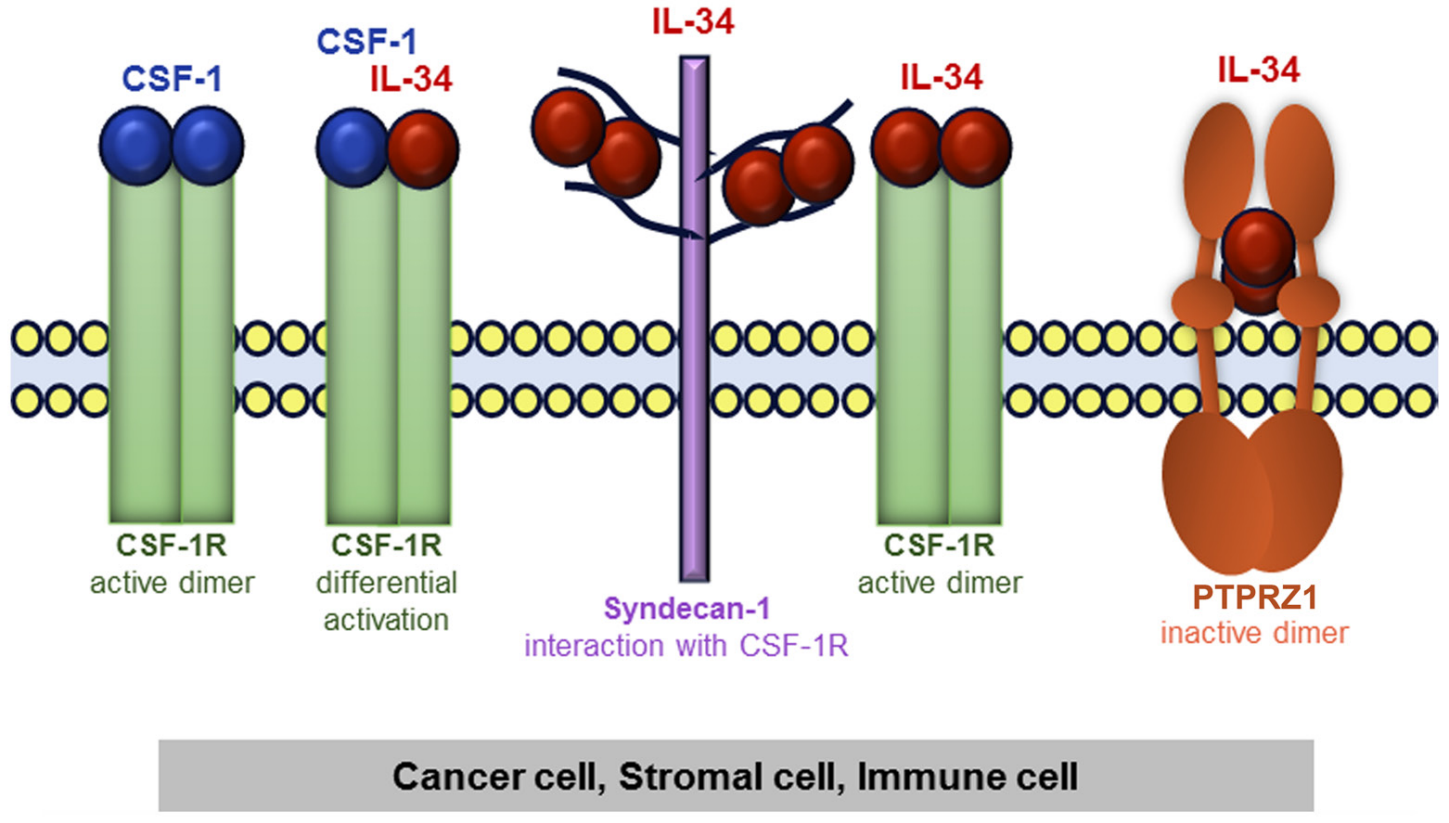
Schematic representation of IL-34 and CSF-1 receptors in tumor tissue consisting of cancer cells, stromal cells, and immune cells IL-34 and CSF-1 bind to CSF-1R. Heteromeric CSF-1/IL-34 can also form, which may differentially regulate activation/localization of CSF-1R. Additionally, IL-34 can bind to syndecan-1, which then regulates CSF-1R activity. Finally, IL-34 also binds to PTPRZ1.

**Figure 5:**
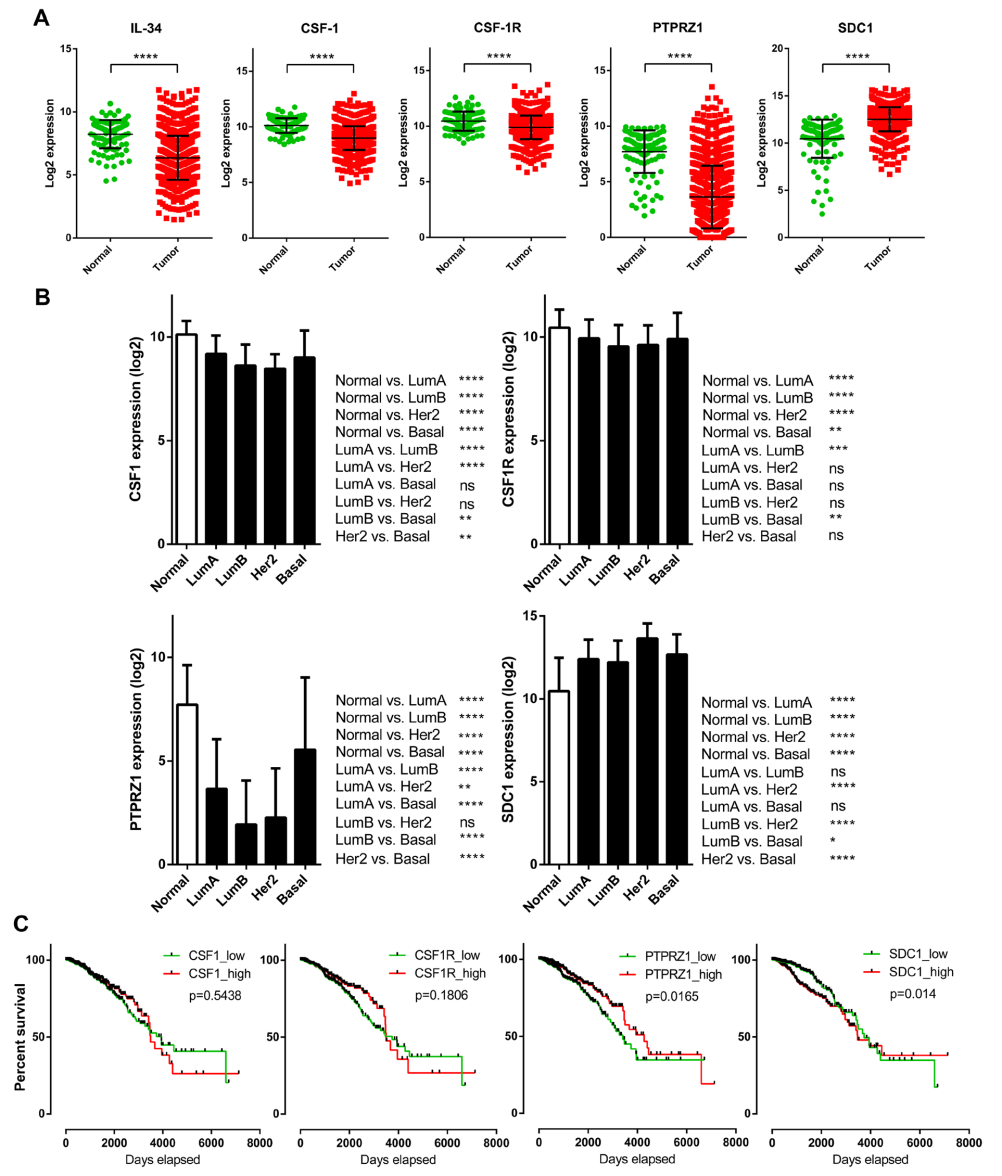
Prognostic significance and tumor-specific changes of expression for the IL-34 system in breast cancer patients **(A)** IL-34, CSF-1, CSF-1R, PTPRZ1, and syndecan-1 expression in TCGA breast cancer samples (n = 1102) vs. normal breast tissue (n = 113). Scatter plots show relative mRNA expression (log2). Each dot represents a single tissue sample. Black lines indicate means and standard deviations. P-values were determined via Mann–Whitney *U* tests (two-sided). **(B)** CSF-1, CSF-1R, PTPRZ1, and syndecan-1 mRNA expression in normal breast tissue and in PAM50 breast cancer subclasses. The bar graph shows the mRNA levels (log2) across normal breast tissue (n = 113) and the molecular subtypes of breast cancer (luminal A, n = 412; luminal B, n = 188; HER2-enriched, n = 64; basal, n = 140) of the TCGA BRCA dataset. Kruskal–Wallis and Dunn’s multiple comparison tests were calculated. Error bars indicate standard deviations. ^*^, *p* < 0.05; ^**^, *p* < 0.01; ^***^, *p* < 0.001; ^****^, *p* < 0.0001. **(C)** Kaplan–Meier plots of overall survival between CSF-1, CSF-1R, PTPRZ1, and SDC1 high and low expressing breast cancer patients (n = 1056) of the TCGA BRCA dataset. Median expression values were used as cut-offs for group separation. Log rank tests were calculated.

There is not necessarily a sole factor mediating the biological activities of IL-34, because, unlike CSF-1, IL-34 binds not only to CSF-1R, but also to PTPRZ1 and syndecan-1 (Figure 4). Thus, we analyzed potential mRNA expression differences of CSF-1, CSF-1R, PTPRZ1, and syndecan-1 between breast cancer samples and normal breast tissue samples from the TCGA breast cancer dataset (Figure 5A). CSF-1 and CSF-1R mRNA expression in tumors (median: 9.02 and 9.97, respectively) was below levels of normal tissue (median: 10.21 and 10.5, respectively). However, the strongest downregulation of gene expression in tumors compared to normal breast tissues was found for PTPRZ1 (median: 3.18 vs. 8.21). In contrast, syndecan-1 mRNA expression was higher in primary breast tumors vs. normal tissue (median: 12.62 vs. 11.05; Figure 5A). All these differences were statistically highly significant. To evaluate if syndecan-1 gene expression is associated with the IL-34 axis, we examined correlations between syndecan-1 and IL-34, CSF-1, CSF-1R, and PTPRZ1 mRNA expression and found a weak positive correlation between syndecan-1 and IL-34 (Pearson correlation r = 0.20, *p* < 0.0001; Supplementary Figure 1, Table 2). In contrast, the Pearson’s correlation coefficients for CSF-1, CSF-1R, and PTPRZ1 were 0.01, 0.09, and 0.06, respectively, indicating a lack of correlation with syndecan-1 (Supplementary Figure 1, Table 2).

**Table 2:** Correlation between SDC1 mRNA and mRNA levels for IL-34, CSF-1, CSF-1R, and PTPRZ1

	IL-34	CSF-1	CSF-1R	PTPRZ1
	r (*p*)	r (*p*)	r (*p*)	r (*p*)
SDC1	**0.20 (<0.0001)**	0.01 (ns)	0.09 (0.003)	0.06 (0.053)

r, Pearson’s correlation coefficient; *p*, *p*-value; ns, not significant. (TCGA BRCA dataset).

Analysis of CSF-1 and CSF-1R expression in molecular breast cancer subtypes revealed generally lower levels for both when compared to normal tissue. Differences in mRNA levels related to normal tissue were more pronounced for PTPRZ1, similar to IL-34. Luminal and HER-2 subtypes showed clearly diminished levels. Basal subtype PTPRZ1 levels were significantly higher in comparison to luminal and HER-2 subtypes, although these patients also express PTPRZ1 at levels below normal PTPRZ1 expression. In contrast, syndecan-1 levels were significantly higher in all molecular subtypes than the respective normal tissue values (Figure 5B).

Next, we examined the prognostic effect of CSF-1, CSF-1R, PTPRZ1, and syndecan-1 expression in the breast cancer TCGA dataset by generating Kaplan–Meier survival curves split in high (top 50%) and low (bottom 50%) gene expression (Figure 5C). In contrast to IL-34, high expression of CSF-1 (log-rank *p =* 0.5438) and CSF-1R (log-rank *p =* 0.1806) was associated with worse outcome during the follow-up period. These data, although not reaching statistical significance, are supported by previous observations showing that high expression of CSF-1/CSF-1R in neoplastic epithelial breast cancer correlates with a poor prognosis [29–31]. On the other hand, high expression of PTPRZ1 was associated with better outcome (log-rank *p =* 0.0165), whereas low expression of syndecan-1 was found to be associated with better outcome (log-rank *p =* 0.014). These data suggest differential expression and survival statistics for the IL-34 receptors CSF-1R, PTPRZ1, and syndecan-1.

**Figure 6:**
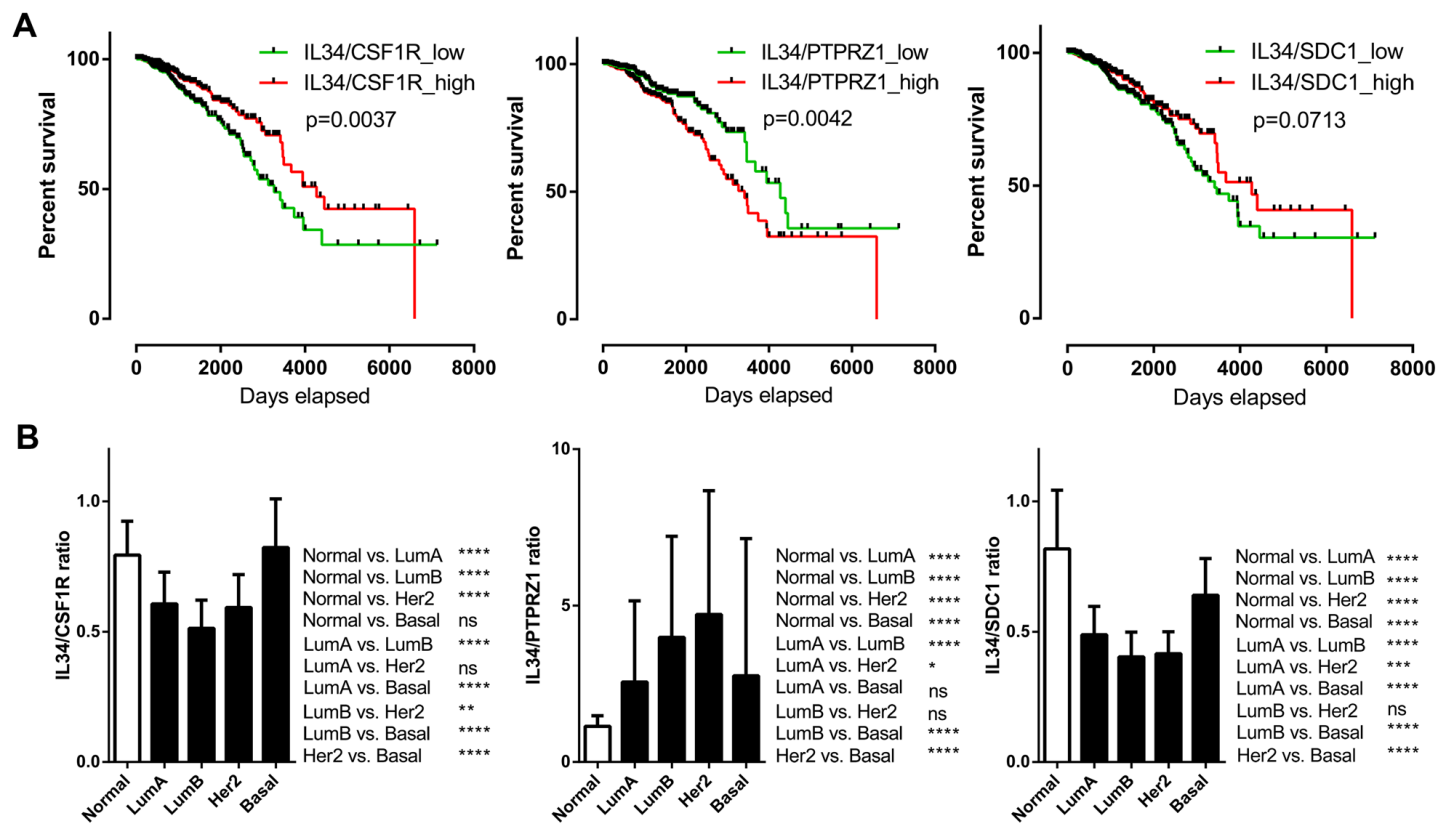
IL-34 ligand-receptor mRNA expression ratio **(A)** Kaplan–Meier plots of overall survival between IL-34/CSF1R, IL-34/PTPRZ1, and IL-34/SDC1 mRNA ratio high and low breast cancer patients (n = 1056) of the TCGA BRCA dataset. Median expression values were used as cut-offs for group separation. Log rank tests were calculated. **(B)** IL-34/CSF1R, IL-34/PTPRZ1, and IL-34/SDC1 mRNA ratio median values in normal breast tissue and in PAM50 breast cancer subclasses. The bar graph shows the IL-34/receptor mRNA ratio across normal breast tissue (n = 113) and the molecular subtypes of breast cancer (luminal A, n = 412; luminal B, n = 188; HER2-enriched, n = 64; basal, n = 140) of the TCGA BRCA dataset. Kruskal–Wallis and Dunn’s multiple comparison tests were calculated. Error bars indicate standard deviations. ^*^, *p* < 0.05; ^**^, *p* < 0.01; ^***^, *p* < 0.001; ^****^, *p* < 0.0001.

We subsequently examined the prognostic effect of IL-34/CSF-1R, IL-34/PTPRZ1, and IL-34/syndecan-1 expression ratios by generating Kaplan–Meier survival curves split in high (top 50%) and low (bottom 50%) IL-34/receptor ratio (Figure 6A). A high IL-34/CSF-1R ratio (log-rank *p =* 0.0037) was associated with better outcome. Likewise, a high IL-34/syndecan-1 ratio showed positive correlation with better survival, but did not reach significance here (log-rank *p =* 0.0713). A high IL-34/PTPRZ1 ratio on the other hand correlated with poor prognosis (log-rank *p* = 0.0042).

Further analysis of IL-34/receptor ratios in molecular subtypes showed that the IL-34/CSF-1R mRNA expression ratio was significantly lower in luminal and HER-2 but not basal subtypes. The IL-34/syndecan-1 ratio was significantly decreased in all molecular subtypes in comparison with normal tissue, while the IL-34/PTPRZ1 mRNA expression ratio was significantly higher in all molecular subtypes (Figure 6B). These results show the complexity of IL-34/receptor mRNA ratios in molecular subtypes of breast cancer.

### IL-34, PTPRZ1, and syndecan-1 are not associated with myeloid cells in the breast cancer microenvironment

Tumors are complex environments, composed of transformed cells as well as stromal cells and immune infiltrates and there is a great deal of evidence that points to the stroma and immune cells as major regulators of tumor progression. We used the ESTIMATE algorithm to correlate the extent of stromal cells with expression of IL-34, CSF-1, CSF-1R, PTPRZ1, and syndecan-1 in breast cancer tissue from the TCGA breast cancer dataset. The stromal score was strongly associated with CSF-1R levels and weakly with CSF-1 and syndecan-1 levels. IL-34 showed only little and PTPRZ1 no association with the stromal score (Supplementary Figure 2, Table 3).

**Table 3:** Stromal score compared with gene expression

	Stromal sc
	r (*p*)
IL-34	**0.20 (<0.0001)**
CSF-1	**0.46 (<0.0001)**
CSF-1R	**0.64 (<0.0001)**
PTPRZ1	**0.09 (0.003)**
SDC1	**0.33 (<0.0001)**

Pearson’s product-moment correlation of stromal cell infiltration and gene expression in the TCGA BRCA dataset. ESTIMATE algorithm was used to correlate the level of infiltrating stromal cells with in breast cancer tissues. Stromal sc, Stromal score; r, Pearson’s correlation coefficient; *p*, *p*-value.

**Figure 7:**
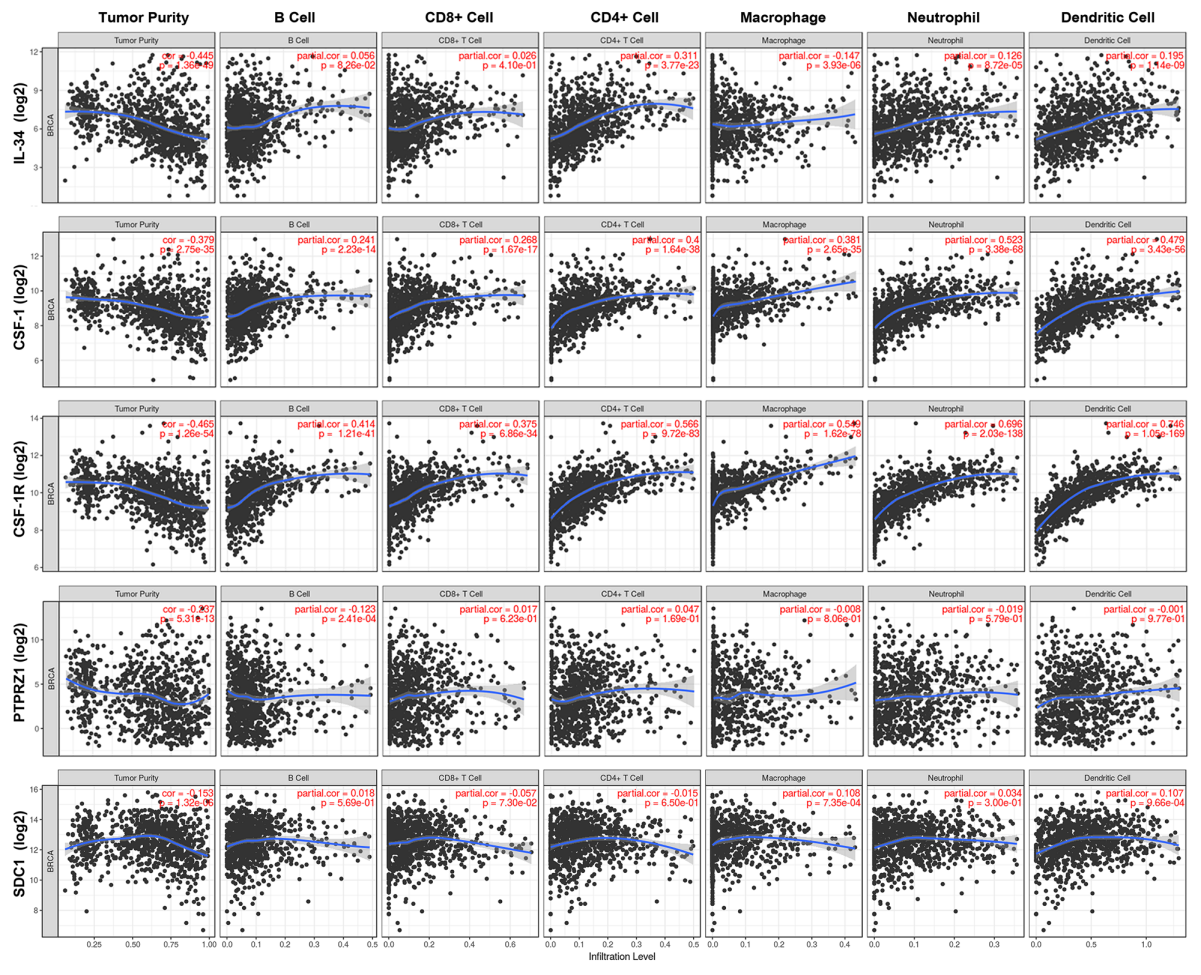
Immune cell landscape of breast cancer compared with TCGA gene expression of *IL-34*, *CSF-1*, *CSF-1R*, *PTPRZ1*, and *SDC1* (syndecan-1) Scatter plots were generated using the online tool TIMER to identify different profiles of immune cells associated with investigated genes. Each dot represents a single tumor sample. (see also Table 5, Supplementary Table 1).

Immune cell subsets present in the microenvironment surrounding cancer cells include B cells, T cells, macrophages, neutrophils, and dendritic cells. We applied the TIMER tool to investigate tumor purity and the infiltrating immune cell landscape of breast cancer (B cells, CD4+ T cells, CD8+ T cells, neutrophils, macrophages and dendritic cells) in the context of mRNA expression of our target genes (Figure 7, Table 4, Supplementary Table 1). Notably, CSF-1 and CSF-1R shared the same immune cell profile, showing a clear association with neutrophils, dendritic cells, macrophages and CD4+ T-cell populations and to a lesser extent with CD8+ T cells and B cells. In contrast, IL-34 even showed a slight negative association with macrophages and little (neutrophils and dendritic cells) or no (B cells, CD8+ T cells) association with other immune cell populations. Only CD4+ T cells exhibited a weak positive correlation with IL-34. Little or no correlation was found between analyzed immune cell populations and both PTPRZ1 and syndecan-1. CSF-1, CSF-1R, and IL-34 were weakly negatively associated with tumor purity, while the lack of association between immune cell infiltration and gene expression of PTPRZ1 and syndecan-1 was also reflected by tumor purity values (Figure 7, Table 4, Supplementary Table 1).

**Table 4:** Immune cell landscape compared with gene expression

	Tumor purity	B cell	CD8+ T cell	CD4+ T cell	Macrophage	Neutrophil	Dendritic cell
	r	r	r	r	r	r	r
IL-34	**--**			**++**	**−**	**+**	**+**
CSF-1	**--**	**+**	**+**	**++**	**++**	**+++**	**++**
CSF-1R	**--**	**++**	**++**	**+++**	**+++**	**+++**	**+++**
PTPRZ1	**−**	**−**					
SDC1	**−**				**+**		**+**

Categorized Pearson’s product-moment correlation of immune cell landscape of breast cancer compared with TCGA gene expression of *IL-34*, *CSF-1*, *CSF-1R*, *PTPRZ1*, and *SDC1* (syndecan-1) (TIMER).

r, categorized Pearson’s correlation coefficient;

(---), −1.0 to −0.5, strong negative association; (--), −0.5 to −0.3, weak negative association; (−), −0.3 to 0.1, little association.

(+), +0.1 to 0.3, little association; (++) +0.3 to +0.5, weak positive association; (+++), +0.5 to +1.0, strong positive association.

These findings suggest that IL-34 and its receptors PTPRZ1 and syndecan-1 are not linked to myeloid cells, whereas the CSF-1/CSF-1R axis shows a strong correlation with myeloid cells in the tumor microenvironment.

### Differing association of macrophage subsets with IL-34 and CSF-1

**Figure 8:**
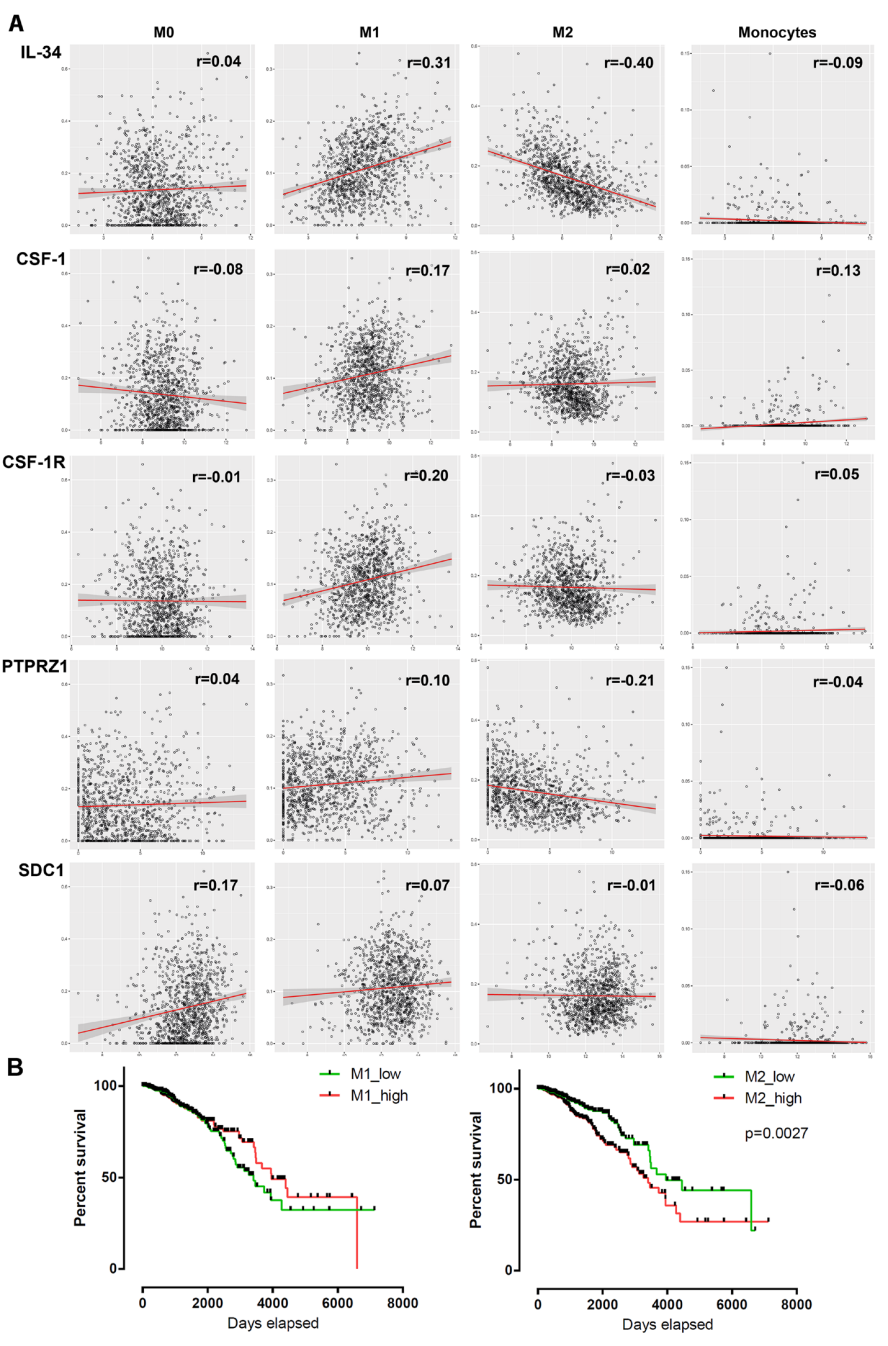
Association between mRNA expression of 5 genes and macrophage infiltration in breast tumors **(A)** Scatter plots show the correlation between *IL-34*, *CSF-1*, *CSF-1R*, *PTPRZ1* and *SDC1* mRNA expression (log2 scale) and M0, M1, M2 and monocyte infiltration scores (obtained from TCIA database) in tumor samples from the TCGA BRCA dataset. Each circle represents a single tumor sample. Regression lines and confidence intervals are shown in red and grey, respectively. **(B)** Kaplan–Meier plots of overall survival between M1/M2 high and low infiltrated breast cancer patients (n = 1052) of the TCGA BRCA dataset. Median macrophage scores from TCIA database were used as cut-offs for group separation. Log rank tests were calculated.

Despite the lack of a significant link between IL-34 and macrophages, we assessed the potential association between macrophage subsets and IL-34, CSF-1 and their receptors. We analyzed tumor-associated monocyte, differentiated M0 macrophage, as well as polarized M1 and M2 macrophage signatures in the breast cancer TCGA dataset using the TCIA database [32]. M0 macrophages are presumed naïve cells that have not been stimulated, whereas M1 or M2 macrophages received signals that promote activation and functional polarization [33]. The comparison revealed a weak positive association of IL-34 and PTPRZ1 with M1 macrophages, while both were weakly negatively associated with M2 macrophages (Figure 8A, Table 5, Supplementary Table 2). Little association with M1 macrophages was found for CSF-1, CSF-1R, and PTPRZ1 and we found no relationship between M2 macrophages and CSF-1, CSF-1R, and syndecan-1 in the dataset. Furthermore, there was little positive correlation of CSF-1 with monocytes and of syndecan-1 with differentiated M0 macrophages. No significant correlation could be identified between the other investigated genes and monocytes or M0 macrophages (Figure 8A, Table 5, Supplementary Table 2).

**Table 5:** Macrophage infiltration scores and stromal score compared with gene expression

	Monocytes	M0	M1	M2	Stromal sc
	r	r	r	r	r
IL-34			**++**	**--**	**+**
CSF-1	**+**		**+**		**++**
CSF-1R			**+**		**+++**
PTPRZ1			**+**	**−**	
SDC1		**+**			**++**

Categorized Pearson’s product-moment correlation correlation between *IL-34*, *CSF-1*, *CSF-1R*, *PTPRZ1* and *SDC1* (syndecan-1) mRNA expression and M0, M1, M2 and monocyte infiltration scores (obtained from TCIA database) in tumor samples from the TCGA BRCA dataset. M0, M0 macrophages; M1, M1 macrophages; M2, M2 macrophages; Stromal sc, Stromal score; r, categorized Pearson’s correlation coefficient;

(---), −1.0 to −0.5, strong negative association; (--), −0.5 to −0.3, weak negative association; (−), −0.3 to 0.1, little association.

(+), +0.1 to 0.3, little association; (++) +0.3 to +0.5, weak positive association; (+++), +0.5 to +1.0, strong positive association.

In addition, we assessed the prognostic association of M1 and M2 macrophages in breast cancer by stratifying our population for M1 and M2 high/low. As expected, high numbers of tumor-associated M2 macrophages were found to predict worse outcomes than pro-inflammatory M1 macrophages (Figure 8B).

These findings indicate varying degrees of the relationship between macrophage subsets and IL-34 and CSF-1.

### M0, M1, and M2 macrophages stratified by PAM50 subtypes

To gain a better understanding of associations between IL-34/CSF-1, their receptors and different macrophage subtypes, we analyzed M0, polarized M1 and M2 macrophage scores from TCIA database stratified by PAM50 subtypes. In addition, we analyzed stromal, immune, and ESTIMATE scores to assess the presence of infiltrating stromal and immune cells in tumor tissue using the TCGA gene expression data.

We observed considerable variation between gene expression, macrophage subsets, and scores, and many of these associations are statistically significant (Table 6, Supplementary Table 3, Supplementary Figure 3). Stratification for M0, M1 and M2 macrophage phenotype yielded a pattern, in which IL-34 expression correlated positively with M1 phenotype in luminal subtypes and was found to be generally negatively correlated with M2 macrophages, with the exception of basal subtype. Only little or no correlations with macrophage subsets were found for PRTPRZ1 expression. On the other hand, syndecan-1 showed a positive relationship was found between syndecan-1 and M0 and M2 macrophages in HER2 subtype, whereas no or little correlations were seen in other subtypes. Unexpectedly, both CSF-1 and CSF-1R expression correlated weakly with M1 macrophages in basal subtype, while no or little correlations were found in other subtypes.

**Table 6:** Macrophage infiltration and stromal cell/immune cell infiltration in breast tumors stratified by PAM50 calls

Luminal A subtype - Categorized Pearson’s product-moment correlation						
	M0	M1	M2	Stromal sc	Immune sc	ESTIMATE sc
	r	r	r	r	r	r
IL-34	**−**	**++**	**---**	**++**	**++**	**+++**
CSF-1			**+**	**++**	**++**	**++**
CSF-1R		**+**		**+++**	**+++**	**+++**
PTPRZ1	**−**	**+**	**--**	**+**		**+**
SDC1	+			**+++**		**++**

Luminal B subtype - Categorized Pearson’s product-moment correlation						
	M0	M1	M2	Stromal sc	Immune sc	ESTIMATE sc
	r	r	r	r	r	r
IL-34		**++**	**--**	**++**	**++**	**++**
CSF-1			**+**	**++**	**+++**	**+++**
CSF-1R		**+**		**+++**	**+++**	**+++**
PTPRZ1						
SDC1		+		**++**	**+**	**++**

HER2 subtype -Categorized Pearson’s product-moment correlation						
	M0	M1	M2	Stromal sc	Immune sc	ESTIMATE sc
	r	r	r	r	r	r
IL-34			**--**		**++**	**++**
CSF-1				**+++**	**+++**	**+++**
CSF-1R				**+++**	**+++**	**+++**
PTPRZ1						
SDC1	++	**−**	++	**++**		

Basal subtype -Categorized Pearson’s product-moment correlation						
	M0	M1	M2	Stromal sc	Immune sc	ESTIMATE sc
	r	r	r	r	r	r
IL-34				**+**	**+**	**+**
CSF-1		**++**	**−**	**+++**	**+++**	**+++**
CSF-1R		**++**		**+++**	**+++**	**+++**
PTPRZ1	+	**−**			**−**	
SDC1						

Association between mRNA expression of 5 genes and macrophage infiltration as well as stromal cell/immune cell infiltration in breast tumors stratified by PAM50 calls from the TCGA BRCA dataset.

M0, M0 macrophages; M1, M1 macrophages; M2, M2 macrophages; Stromal sc, Stromal score; Immune sc, Immune score; Estimate sc, Estimate score. r, categorized Pearson’s correlation coefficient;

(---), −1.0 to −0.5, strong negative association; (--), −0.5 to −0.3, weak negative association; (−), −0.3 to 0.1, little association.

(+), +0.1 to 0.3, little association; (++) +0.3 to +0.5, weak positive association; (+++), +0.5 to +1.0, strong positive association.

M0, M1, M2 (TCIA database); Stromal sc., Immune sc., Estimate sc. (ESTIMATE database).

By comparing stromal, immune and ESTIMATE scores in intrinsic breast cancer subtypes, we identified strong relationships of CSF-1 and CSF-1R to all scores (Table 6, Supplementary Table 3, Supplementary Figure 4). In contrast, correlation of IL-34 was weakly positive in luminal A and B subtypes, got lower in HER2 subtype and was lowest in basal subtype. A similar relationship was found between intrinsic subtype and syndecan-1. Syndecan-1 correlated positively with stromal and ESTIMATE score in luminal A and B subtypes, with stromal score in HER2 subtype, but no correlation was observed in basal subtype. However, syndecan-1 correlated only little or not with immune score in all subtypes. PTPRZ1 shows little or no correlation with any of these scores in all subtypes (Table 6, Supplementary Table 3, Supplementary Figure 4).

Together, these data demonstrate a subtype specific correlation pattern between IL-34, its receptors and macrophage subtypes. They also show a differential relationship between gene expression and stromal, ESTIMATE, and immune scores in intrinsic subtypes. Notably, IL-34 and its receptors PTPRZ1 and syndecan-1 showed no relationship to the analyzed scores in basal subtype.

### IL-34 and CSF-1 differentially regulate breast cancer cell migration and signaling

To experimentally evaluate, whether IL-34 and CSF-1 directly regulate breast cancer cells depending on their subtype, we assessed the effect of recombinant IL-34 and CSF-1 on luminal-like MCF7, HER-2-positive SK-BR-3, and basal type MDA-MB-231 breast cancer cells.

**Figure 9:**
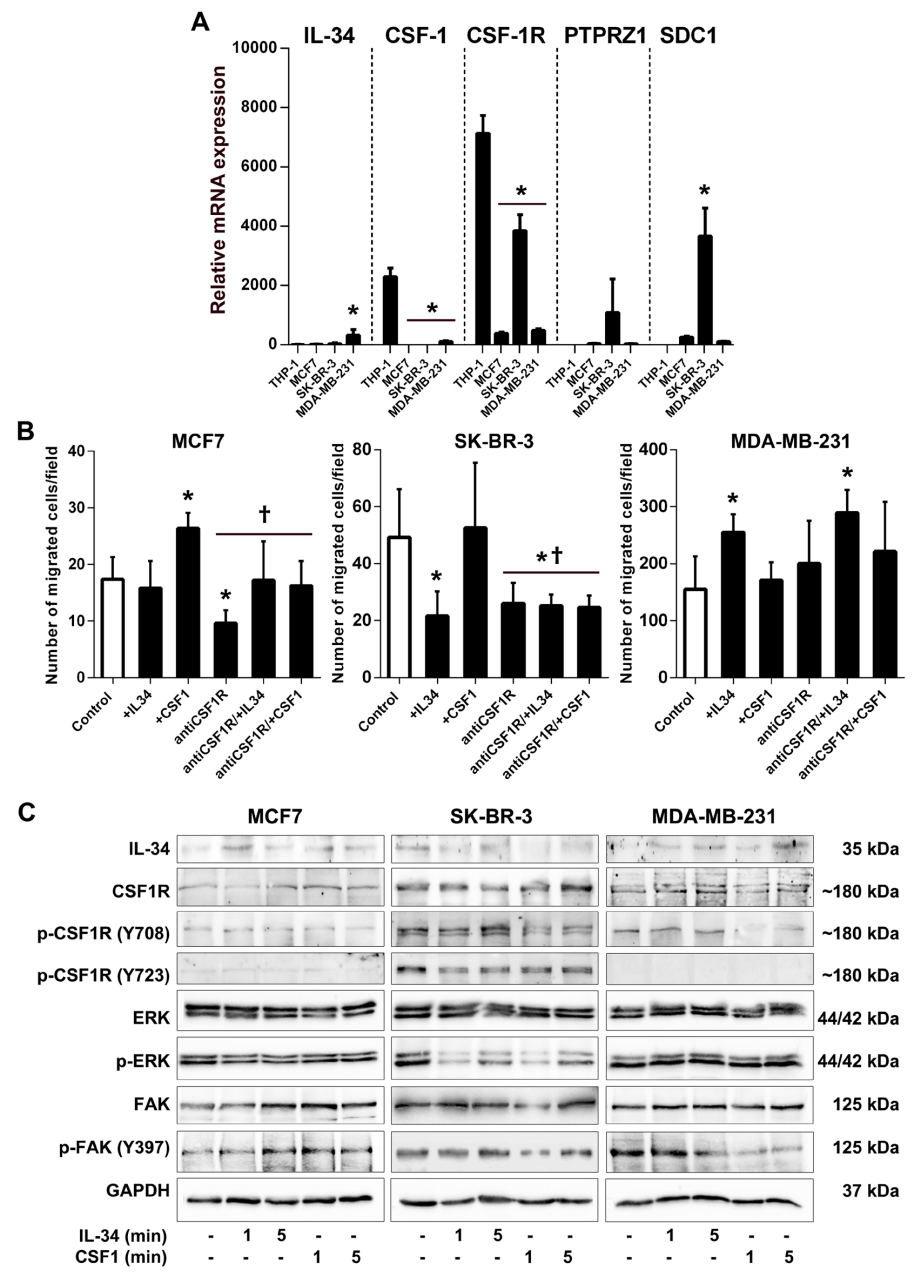
IL-34 differentially regulates migration and signaling of human breast cancer cell lines **(A)** Comparison of gene expression in MCF7, SK-BR-3, and MDA-MB-231 breast cancer cells with THP-1 macrophages. Graphs show results of qRT-PCR for *IL-34*, *CSF-1*, *CSF-1R*, *PTPRZ1* and *SDC1* performed on RNA from human MCF7, SK-BR-3 and MDA-MB-231 breast cancer cells as well as THP-1 macrophages. **(B)** Quantification of migrated MCF7, SK-BR-3 and MDA-MB-231 breast cancer cells from an *in vitro* migration assay are shown. Cells were either left unstimulated (control) or stimulated with IL-34, CSF-1 or pretreated with CSF1-R blocking antibody (antiCSF1R) prior to cytokine treatment. ^*^, *p* < 0.05 vs. control;, *p* < 0.05 vs recCSF1. **(C)** Differential regulation of signaling upon IL-34 or CSF-1 treatment in human breast cancer cell lines. MCF7, SK-BR-3, and MDA-MB-231 breast cancer cells were stimulated with recombinant IL-34 or CSF-1 protein for the indicated times. Western blot images of indicated proteins in breast cancer cells are shown. p- indicates phosphorylated proteins.

First, we compared *IL-34*,*CSF-1*, *CSF-1R*, *PTPRZ1*, and *SDC1* gene expression between human MCF7, SK-BR-3 and MDA-MB-231 cancer cells in comparison to human THP-1 macrophages. Quantitative RT-PCR showed that IL-34 was expressed at low levels in all cell lines. A high CSF-1 mRNA level was only found in THP-1 macrophages. CSF-1R mRNA expression levels were much higher in THP-1 and SK-BR-3 cells as compared to MCF-7 and MDA-MB-231 cells. Notably, PTPRZ1 and syndecan-1 mRNA was expressed at high levels in SK-BR-3 cells, while only low (MCF7 and MDA-MB-231 cells) or no expression (THP-1 cells) was found in other cell lines (Figure 9A). Together, these data indicate that breast cancer cells have a varying potential to respond to IL-34.

IL-34 is known to increase migration of monocytes and macrophages [22]. Here, we set out to evaluate the effects of IL-34 and CSF-1 on cancer cell migration in transwell assays. Migration of MCF7 cells was not affected by IL-34, whereas IL-34 stimulation significantly decreased migration of SK-BR-3 cells by 56%. In contrast, IL-34 enhanced the ability of MDA-MB-231 cells to migrate by 64% relative to control cells. The effect of CSF-1 was quite different. CSF-1 stimulation significantly enhanced cell migration in MCF-7 breast cancer cells by 52%, while no significant increases were observed for SK-BR-3 (7%; n.s) and MDA-MB-231 (10%; n.s) relative to control cells (Figure 9B).

To address the role of CSF-1R in cancer cell migration, we pretreated cells with a CSF-1R blocking antibody (Figure 9B). CSF-1R blockade inhibited CSF-1-induced migration in MCF-7 cells by 45%. We also show that IL-34 inhibited SK-BR-3 migration independent of the CSF-1R as shown in cells treated with the CSF-1R blocking antibody. This finding suggests that IL-34 inhibits migration through the PTPRZ1 receptor, consistent with expression of PTPRZ1 and CSF-1R on SK-BR-3 cells. Furthermore, IL-34 increased migration of MDA-MB-231 cells in the presence of a blocking antibody against CSF-1R by 87%, also suggesting a CSF-1R-independent mechanism in this cell line (Figure 9B).

These findings demonstrate that IL-34 can promote or inhibit breast cancer cell migration depending on IL-34-receptor expression profile and molecular subtype.

### Signal activation by IL-34 and CSF-1 exhibit differences in human breast cancer cells

To clarify the signaling basis by which IL-34 and M-CSF may differently regulate breast cancer cells, we investigated signaling events triggered by IL-34 and CSF-1.

When analyzed by Western blotting, we found no obvious difference in the strength of CSF-1R, ERK, and FAK activation between IL-34- and CSF-1-stimulated MCF-7 breast cancer cells (Figure 9B). However, we found obvious differences in protein phosphorylation between IL-34- and CSF-1-stimulated SK-BR-3 and MDA-MB-231 cells (Figure 9C). It has been shown that CSF-1R rapidly phosphorylates its own tyrosine residues Tyr708 and Tyr723 when activated by IL-34, which triggers the interaction of the activated receptor with downstream signaling molecules [22]. IL-34 strongly phosphorylated tyrosine residue Tyr708 of the CSF-1R in SK-BR-3 cells, whereas Tyr708 phosphorylation remained unchanged by IL-34 in MDA-MB-231 cells. On the contrary, the Tyr708 signals declined rapidly in SK-BR-3 and MDA-MB-231 cells treated with CSF-1. Phosphorylation of tyrosine residue Tyr723 of the CSF-1R declined by IL-34 and CSF-1 in SK-BR-3 cells, while no Tyr723 phosphorylation was found in MDA-MB-231 and MCF-7 cells. We also observed differences in extracellular signal-regulated kinase (ERK) phosphorylation between cell lines (Figure 9C). We show that both CSF-1 and lL-34 triggered ERK phosphorylation in MDA-MB-231 cells, whereas a rapid and strong decline of ERK phosphorylation was observed in SK-BR-3 cells.

Phosphorylation of focal adhesion kinase (FAK) by IL-34 and CSF-1 has also been reported [34]. Importantly, a strong and rapid decline of focal adhesion kinase (FAK) phosphorylation at residue Tyr397 by CSF-1 but not IL-34 was observed in MDA-MB-231 cells, whereas FAK phosphorylation increased by IL-34 and CSF-1 treatment in SK-BR-3 cells (Figure 9C).

Collectively, these results indicated that IL-34 and CSF-1 differentially activated signaling pathways in three breast cancer cell lines representing luminal, HER-2 and basal intrinsic subtypes.

## DISCUSSION

IL-34 has received much attention as a newly discovered member of the interleukin family, which shares the CSF-1R with CSF-1 [12]. However, while the role of CSF-1/CSF-1R has been extensively studied in breast cancer, the implication of IL-34 in breast cancer formation and progression is still poorly understood.

In this article, correlative analysis of IL-34 expression and breast cancer prognosis stratified by PAM50 tumor subtype showed an unexpected prognostic relationship between intrinsic subtype and overall survival that demonstrated patterns contrasting previous studies analyzing CSF-1 and CSF-1R. Specifically, high IL-34 levels were linked to better prognosis in luminal and HER2 subtypes, but to poorer prognosis in the basal subtype. The finding that high levels of IL-34 are associated with better prognosis in certain breast cancer subtypes is surprising, since the expression of CSF-1 and the common receptor CSF-1R has been linked to aggressive behavior and poor prognosis in breast cancer patients[29, 35–37]. CSF1 recruits macrophages to the tumor site on the one hand [37] and CSF-1-educated TAMs have a central role in supporting tumor cell survival, proliferation, motility and in suppressing anti-tumor immunity on the other [38]. Characterization of macrophage phenotypes in breast cancer subtypes using CIBERSORT_LM22 values and the TCIA database showed, as expected, that CSF-1/CSF-1R expression was positively associated with macrophages in all subtypes in the analyzed dataset.

Quite on the contrary, analysis of the immune cell landscape revealed even a slight negative correlation between IL-34 expression and macrophages. Broken down by molecular subtype and macrophage phenotypes revealed that high IL-34 expression correlated negatively with immunosuppressive M2 and positively with M1 polarized tumor suppressor macrophages in luminal subtypes, whereas no correlation with macrophages was found in HER2 and basal subtypes. These findings suggest that the functional role of IL-34 is not coupled to TAMs with an M2-like phenotype in breast cancer. However, it must be taken into account that IL-34 and CSF-1 were associated with different infiltrating immune cell subsets. Varying composition of the infiltrating immune cell subsets in turn may induce a diversity of TAM subtypes, different to the conventional M1/M2 phenotypes [39]. Likewise, as a result of tumor cell heterogeneity distinct populations of TAMs with different phenotypes and functions can be found in the same tumor [40].

Additionally, cancer cells may consume IL-34 in certain subtypes at the same time, since CSF-1/CSF-1R but not IL-34 were strongly associated with stromal- and immune-score in HER2 and basal subtypes. In this regard, the observed differences in the IL-34/CSF-1R ratio may play a role in both, myeloid and cancer cells. *In vitro* experiments in luminal and basal type breast cancer cells revealed that IL-34 and CSF-1 differentially regulate cancer cell migration dependent on the molecular subtype. IL-34 promoted cancer cell migration in basal MDA-MB-231 breast cancer cells independent of the CSF-1R, while it reduced cancer cell migration in HER2-positive SK-BR-3 cells. In contrast, IL-34 had no effect on luminal MCF-7 cancer cell migration, which was regulated by CSF-1/CSF-1R. These findings are in favor of the hypothesis that cancer cells are directly affected by IL-34 signaling via alternative IL-34 receptors.

Consequently, the biology of IL-34 is complex and other reports also suggest that its actions are not expected to be identical to CSF-1/CSF-1R signaling [34, 41]. IL-34 shares very little homology with CSF-1 and has a higher affinity to the CSF-1R [12, 42]. Even more important, binding of IL-34 to PTPRZ1 [21] and syndecan-1 [22] through low affinity interactions with chondroitin sulphate chains demonstrates that the CSF-1R is not mediating all the actions of IL-34.

PTPRZ1 is a member of the receptor tyrosine phosphatase family [21, 43, 44]. Activation of PTPZ leads to increased tyrosine phosphorylation of several transduction pathways and is upregulated in many human cancers, such as lung and prostate cancers, and regulates their proliferation and metastasis [15]. Noteworthy, IL-34 selectively bound PTPRZ1 in CSF-1R-deficient U251 human glioblastoma cells and led to an increase in the tyrosine phosphorylation of FAK and suppression of cell motility [21]. Fittingly, in HER2-positive SK-BR-3 cells IL-34 stimulation increased FAK phosphorylation and decreased cell migration. On the other hand, in basal MDA-MB-231 cells, IL-34 decreased FAK phosphorylation and increased cell migration independent of the CSF-1R. Moreover, only little (luminal A) or no correlation was found for PRTPRZ1 expression with stromal and ESTIMATE scores and also no positive correlation with the immune score, which is consistent with an additional action of IL-34 via the PRTPRZ1 on cancer cells. Together with our *in vitro* experiments these findings suggest that CSF-1R-independent actions of IL-34 via PTPRZ1 should be considered in evaluating IL-34 roles in breast cancer subtypes.

Unlike PTPRZ1, syndecan-1 correlated with stromal score in all subtypes with the exception of basal subtype. Interestingly, a recent report showed that stromal syndecan-1 expression was associated with positive ER status, whereas epithelial syndecan-1 expression was associated with negative ER status [45]. However, reports on stromal syndecan-1 expression and prognosis in breast cancer are controversial [27, 45, 46] and studies from an *in vitro* breast cancer model have suggested that syndecan-1 even directly participates in tumor cell spreading and adhesion[47]. Another report showed that IL-34 also induced the migration of myeloid cells in a syndecan-1-dependent manner [22]. To complicate matters further, we found differential IL-34/syncdecan-1 ratios in molecular subtypes. This is important because the level of syndecan-1 has been suggested to regulate the interaction between IL-34 and the CSF-1R [22]. Importantly, syndecan-1 was not or only weakly associated with macrophages and immune score in our analysis. A link to M0 and M2 macrophages in the HER2 subtype was the only noticeable link observed. Moreover, analysis of macrophage phenotypes showed that the correlation patterns of IL-34 and syndecan-1 were quite different in all molecular subtypes. Thus, we assume that syndecan-1 and IL-34 do not act in concert to regulate macrophages in breast cancer.

Calculation of the ESTIMATE score, to infer tumor purity, and of the stromal score revealed that CSF-1 and CSF-1R are strongly linked to the stromal compartment in all subtypes. In contrast, association of IL-34 with stromal score was confined to luminal subtypes, while PTPRZ1 was not associated with this score in any subtype. Together with the observed low CSF-1R activation in breast cancer cells, we hypothesize that IL-34 and its receptor PTPRZ1 directly regulate cancer cells, whereas CSF-1/CSF-1R are primarily involved in regulation of stromal and immune cells.

In support of this, we observed considerable differences in the composition of the immune infiltrate between breast cancer subtypes by comparing immune cell infiltration with gene expression using the online tool TIMER. CSF-1/CSF-1R were strongly associated with myeloid cell (macrophages, neutrophils, dendritic cells) infiltration. This contrasts sharply with the weak or absent correlation between tumor-infiltrating myeloid cells and expression of IL-34, PTPRZ1, and syndecan-1. An interesting observation in this context is that higher levels of tumor-infiltrating myeloid cells contribute to increased malignancy and correlate with poorer survival [48, 49]. Strikingly, the CSF-1R is not only expressed by macrophages, but also by dendritic cells (DCs), in Langerhans cells, B cells, and to some extent granulocytes including neutrophils (see [15] and references therein). Moreover, neutrophils can produce large amounts of CSF-1 [50]. In contrast to CSF-1, IL-34 is not expressed by myeloid cells and, consequently, has no autocrine role in this context [39], providing further support for a differential role of CSF-1 and IL-34 in myeloid cells. Gentles et al. previously reported neutrophils to be prognostic in breast cancer [49] and a larger proportion of neutrophils was associated with poor outcome in ER+/HER2+ and ER-negative tumors [48]. Recently, neutrophils were shown to promote metastasis through immunosuppression in a model of breast cancer [51]. Importantly, growing evidence also shows that the breast cancer tumor microenvironment can change the phenotype of dendritic cells, transforming them into immunosuppressive DCs, which limit the activity of effector T cells and supports tumor growth and progression [52, 53]. A recent analysis of immune cell subsets in breast cancer has found that activated dendritic cells were strongly associated with poor outcome in ER+/HER2+ tumors [48]. Furthermore, CSF-1/CSF-1R also showed a higher correlation with tumor infiltrating lymphocytes (TILs) than IL-34, PTPRZ1, and syndecan-1. High numbers of TILs can be found in triple negative and HER2-positive tumors [54]. Together, these findings could, at least partly, explain the observed favorable outcome associated with IL-34.

In conclusion, our study shows that the mRNA expression pattern of IL-34 was distinct from CSF-1 and associated with a favorable prognosis dependent on the molecular breast cancer subtype. In addition, gene expression patterns of CSF-1 and CSF-1R but not IL-34 were associated with myeloid cell infiltration. These findings suggest different functions of IL-34 and CSF-1 in breast cancer.

## MATERIALS AND METHODS

### Cell lines

Our own data set of breast cancer cell lines included 14 cell lines derived from carcinomas: AU565, BT-474, CAL-51, CAMA-1, HCC1143, HCC1937, Hs 578T, MCF7, MDA-MB-231, MDA-MB-453, MDA-MB-468, SK-BR-3, T-47D, ZR-75-1. HMEC and Hs 578Bst represent normal mammary tissue, while MCF 10A and MCF 10F were derived from a fibrocystic disease. All cell lines except HMEC were purchased from American Type Culture Collection (ATCC, Manassas, VA, USA) or “Deutsche Sammlung von Mikroorganismen und Zellkulturen” (DSMZ), and were cultivated at 37°C, 5% CO_2_, and 100% humidity. Finite-lifespan untransformed human mammary epithelial cells (HMEC) were kindly provided by M. R. Stampfer [55] and grown in MEGM medium. The human monocytic leukemia cell line THP-1 was cultured in RPMI1640 (Lonza, Basel, Switzerland) medium supplemented with 10% FBS (Life Technologies, Carlsbad, CA, USA) and 2-mercaptoethanol (Sigma-Aldrich, St. Louis, MO, USA) to a final concentration of 0.05 mM. For differentiation of THP-1 cells into mature macrophages, THP-1 cells were cultured in growth medium with 150 nM phorbol 12-myristate 13-acetate (PMA) (Sigma-Aldrich) for 48 h [56].

### Study population

Our own data set of clinical samples included 75 breast cancer cases of Caucasian background from a study population described in detail in [57]. The study was approved by the institutional review board of the Medical University of Vienna (MUV), Vienna, Austria. For those patients who had undergone surgery before the onset of the study, a waiver of specific informed consent was approved by the IRB.

### Quantitative real-time RT-PCR (qRT-PCR)

Isolation of total RNA with TRIreagent (Sigma), quality control with the Bioanalyser 2100 (Agilent, Santa Clara, CA, USA), and reverse transcription with the high-capacity cDNA archive Kit (Applied Biosystems, Foster City, CA, USA) have been described [57]. Each sample was analyzed in duplicate by quantitative reverse transcription-PCR (qRT-PCR) on an Applied Biosystems 7500 fast real-time PCR instrument, using gene-specific primers and fluorescent probes obtained from Applied Biosystems: IL-34, Hs01050926; β-actin, Hs99999903. The mRNA levels of IL-34 were normalized to those of β-actin in each sample. All relative IL-34 mRNA expression levels are presented as log2 values.

For *in vitro* assays, MCF7, SK-BR-3 and MDA-MB-231 breast cancer cells were processed for PCR as described [58]. The primer sequences for analyzed factors are as follows (sense/antisense): IL-34: 5′-AATCCGTGTTGTCCCTCTTG-3′/5′-CAGTACAGCAGCTCCATGACC-3′; CSF-1: 5′-GCTGTTGTTGGTCTGTCTC-3′/5′-CATGCTCTTCATAATCCTTG-3′; CSF-1R: 5′-CTGAGCAAGACCTGGACAAGG-3′/5′-TGCTGTTCACCAGGATGCCAG-3′; PTPRZ1: 5′-TCTACTGCTTTGATGCGGACC-3′/5′-ACGACTAACACTTTCGACTCC-3′; SDC1: 5′- GGGACTCAGCCTTCAGACAG-3′/5′-CTCGTCAATTTCCAGGAGGA-3′. LCDA Version 3.5.3 (Roche, Mannheim, Germany) was used for PCR data analysis. Relative quantification of the signals was performed by normalizing the signals of the different genes to β2-microglobulin as described [58].

### Migration assay

MCF7, SK-BR-3 and MDA-MB-231 breast cancer cell lines were starved over night in serum-free DMEM medium. Breast cancer cells (1×10^5^) in DMEM with 2% FBS were added to the top of each Boyden migration chamber (8 μm, 12-well plate format; BD Biosciences, Palo Alto, CA) with or without CSF-1R-blocking antibody (10μg/ml) followed by treatment with 200 ng/ml recombinant IL-34 or CSF-1 protein (R&D Systems, McKinley Place, MN, USA). After 48 h, medium was removed and membranes were processed as described [59].

### Protein isolation and Western blotting

Breast cancer cell lines were starved for 24 h, one portion of cells was left untreated and one portion of cells was incubated with 200 ng/ml recombinant IL-34 or CSF-1 protein for 1 or 5 min. Cell lysates were prepared as described [60] and 25 μg/lane were separated by 8/14% SDS-PAGE prior to electrophoretic transfer onto Amersham Protan Supported 0.2 μm Nitrocellulose membrane (GE Healthcare, Buckinghamshire, UK). The blots were probed with antibodies against IL-34 (Thermo Fisher Scientific, Waltham, MA, USA), CSF-1R (R&D Systems, McKinley Place, MN, USA), phospho-CSF-1R(Tyr708), phospho-CSF-1R(Tyr723), ERK1/2, phospho-44/42 ERK1/2(Thr202/Tyr204), FAK and pFAK(Tyr397) (Cell Signaling Technology, Danvers, MA, USA) before incubation with horseradish peroxidase–conjugated secondary antibodies (GE Healthcare). GAPDH-HRP staining (Sigma) was used as loading control. Proteins were immunodetected by chemiluminescence (Ace Glow, Peqlab, Erlangen, Germany), scanned using FUSION-FX7 (Vilber Lourmat, Marne-la-Vallée, France) and quantified by Fusion-CAPT-Software 16.07 (Vilber Lourmat).

### Publically available datasets

Level 3 normalized IlluminaHiSeq RNA-sequencing (RNA-seq) data and clinical data from the “The Cancer Genome Atlas” (TCGA) BRCA (breast cancer) dataset were obtained from the Cancer Browser database (https://genome-cancer.ucsc.edu). mRNA expression values of the genes *IL-34, CSF-1, CSF-1R, PTZRZ1* and *SDC1* were extracted from the expression matrix. Overall, mRNA expression data of 1102 breast cancer samples and 113 normal breast tissue samples were available. Information regarding OS was available for 1056 patients. PAM50 calls (luminal A, luminal B, HER2, basal-like and normal-like) were available for 826 patients. Because of the low number, normal-like tumors were excluded from analyses.

### Correlation of mRNA expression and immune cell infiltration

mRNA expression data of *IL-34, CSF-1, CSF-1R, PTZRZ1* and *SDC1* in TCGA BRCA tumor samples were correlated with tumor infiltration of 6 immune cell types (B cells, CD4+ T cells, CD8+ T cells, neutrophils, macrophages and dendritic cells) using the online tool TIMER (Tumor IMmune Estimation Resource; https://cistrome.shinyapps.io/timer/; [61, 62]). In addition, information about tumor purity was obtained from TIMER.

For a more detailed analysis regarding macrophage infiltration and mRNA expression of the 5 genes in the TCGA BRCA dataset, CIBERSORT_LM22 values for monocytes and M0, M1 and M2 macrophages were obtained from “The Cancer Immunome Database” (TCIA, https://tcia.at/home, [32]). These values were then merged with RNA-seq data of the tumor samples. Overall, TCIA scores and RNA-seq data were available for 1091 breast cancer samples.

### ESTIMATE scores (Tumor Purity, Stromal score, Immune score)

ESTIMATE (Estimation of STromal and Immune cells in MAlignant Tumor tissues using Expression data) was used as a resource to get information about tumor purity (proportion of cancer cells in the tumor), and the presence of infiltrating stromal and immune cells in breast cancer tissues from TCGA BRCA gene expression data [63]. This algorithm is based on single sample Gene Set Enrichment Analysis and generates three scores: a stromal score (that captures the presence of stroma in tumor tissue), an immune score (that represents the infiltration of immune cells in tumor tissue), and the ESTIMATE score (that infers tumor purity). ESTIMATE scores for 1100 TCGA BRCA samples were obtained from http://bioinformatics.mdanderson.org/estimate/ and merged with RNA-seq data. Overall, ESTIMATE data and RNA-seq data were available for 1091 breast cancer samples.

### Statistical analysis

Statistical analyses were performed with Statistical Package for the Social Sciences (SPSS) software (version 22.0, SPSS Inc., Chicago, IL, USA) and R version 2.15.1 (“Roasted Marshmallows”), an open-source language and environment for statistical computing [32]. Differences between the indicated groups with respect to relative IL-34 mRNA levels were analyzed by unpaired, two-sided *t*-tests or one-way analysis of variance (ANOVA) with post hoc tests. Kaplan–Meier survival curves were used to estimate OS, DFS, and MFS and the survival differences between high-expression and low-expression groups were assessed by log-rank tests as described [64]. All results were considered statistically significant when *p* values were less than 0.05. All statistical tests were two-sided.

Statistical analyses of TCGA data were performed using R version 3.2.1 and GraphPad Prism 6 software (GraphPad Software Inc., La Jolla, CA, USA). Data normality was tested using the Shapiro–Wilk test. Differences between 2 groups were calculated by unpaired, two-sided Mann–Whitney *U* tests and differences between >2 groups were calculated by Kruskal–Wallis and Dunn’s multiple comparison tests. Survival curves were plotted using the Kaplan–Meier method and compared using log-rank test. Correlation analyses were performed using the *cor.test* function of R and visualized using the *ggplot2* package. All results were considered statistically significant when *p* values were less than 0.05. All statistical tests were two-sided.

## SUPPLEMENTARY MATERIALS FIGURES AND TABLES


