# Supplementary material for: Differential prognostic impact of interleukin-34 mRNA expression and infiltrating immune cell composition in intrinsic breast cancer subtypes
Source: Oncotarget. 2018 May 1;9(33):23126–48. doi: 10.18632/oncotarget.25226 (PMC5955405; doi:10.18632/oncotarget.25226)
Supplement: Supplementary file 1 [file oncotarget-09-23126-s001.pdf]

Differential prognostic impact of interleukin-34 mRNA expression and infiltrating immune cell composition in intrinsic breast cancer subtypes

SUPPLEMENTARY MATERIALS

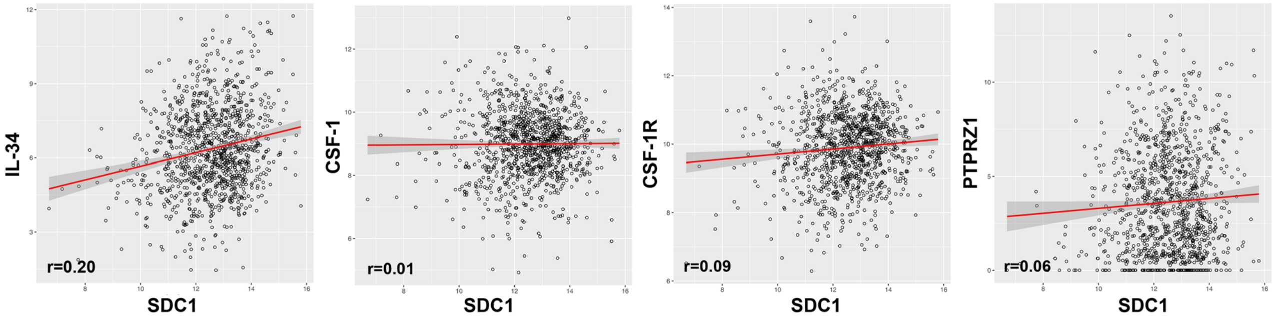

Supplementary Figure 1: Syndecan-1 expression correlations. Scatter plots showing the correlation between SDC1 (syndecan-1) mRNA and mRNA levels for IL-34, CSF-1, CSF-1R, and PTPRZ1 in the TCGA BRCA dataset (log2 scale). Significant correlation only between IL-34 and SDC1. Pearson coefficient (r) is presented.

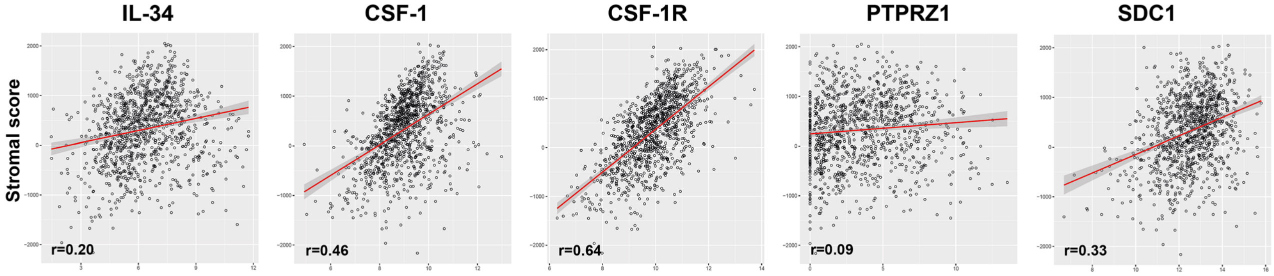

Supplementary Figure 2: Correlation of stromal cell infiltration and gene expression in the TCGA BRCA dataset. ESTIMATE algorithm was used to correlate the level of infiltrating stromal cells with expression of IL-34, CSF-1, CSF-1R, PTPRZ1, and SDC1 (syndecan-1) in breast cancer tissues (n = 1091). Each circle represents a single tumor sample. Regression lines and confidence intervals are shown in red and grey, respectively. X-axis: mRNA expression value (log2), Y-axis: stromal score.

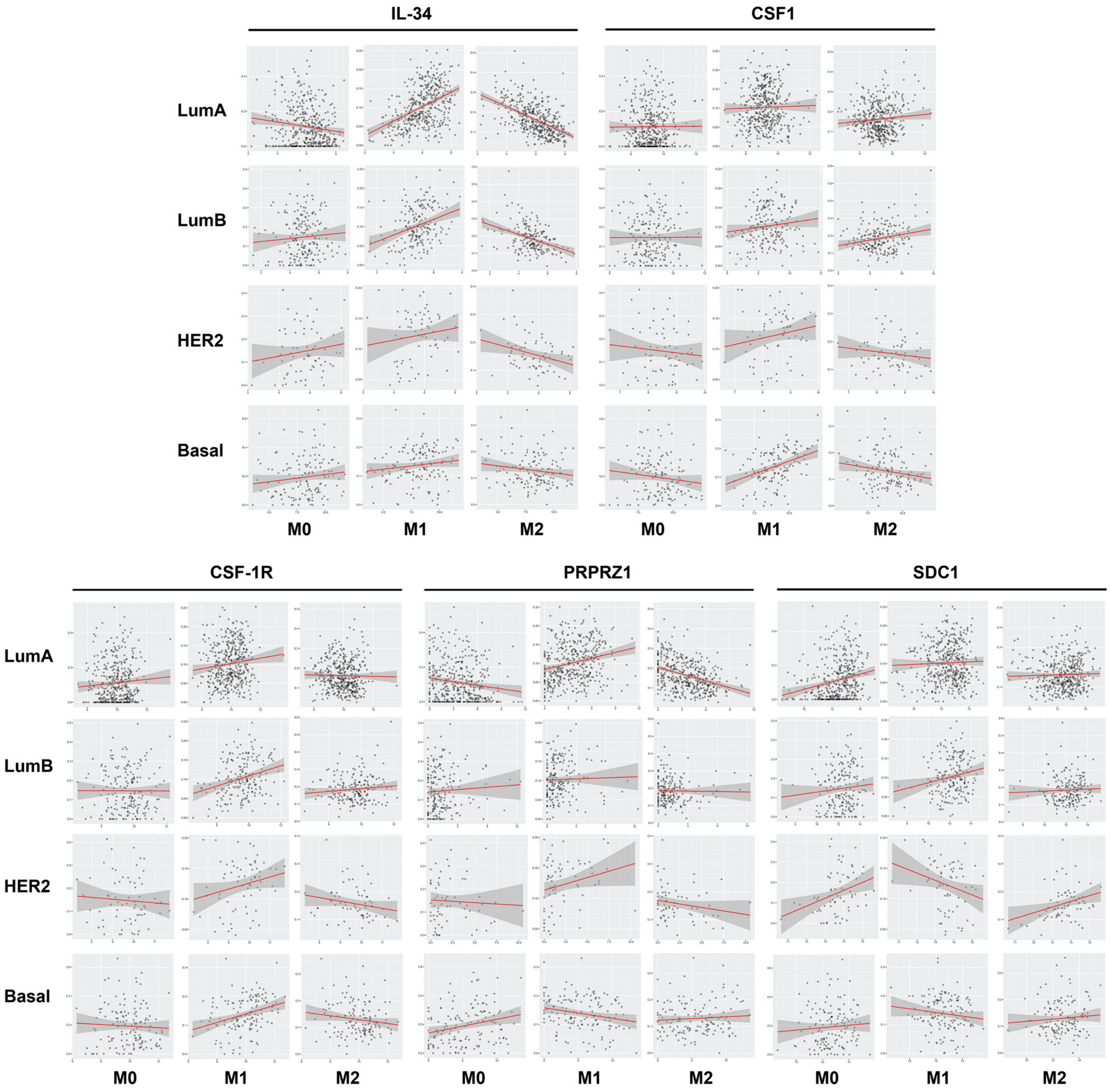

Supplementary Figure 3: Association between mRNA expression of 5 genes and macrophage infiltration in breast tumors stratified by PAM50 calls. Scatter plots demonstrate the correlation between *IL-34*, *CSF-1*, *CSF-1R*, *PTPRZ1* and *SDC1* mRNA expression (log2 scale) and M0, M1, M2 and monocyte infiltration scores (obtained from TCIA database) in luminal A (LumA), luminal B (LumB), HER2-enriched and basal-like (Basal) tumor samples from the TCGA BRCA dataset. Each circle represents a single tumor sample. Regression lines and confidence intervals are shown in red and grey, respectively.

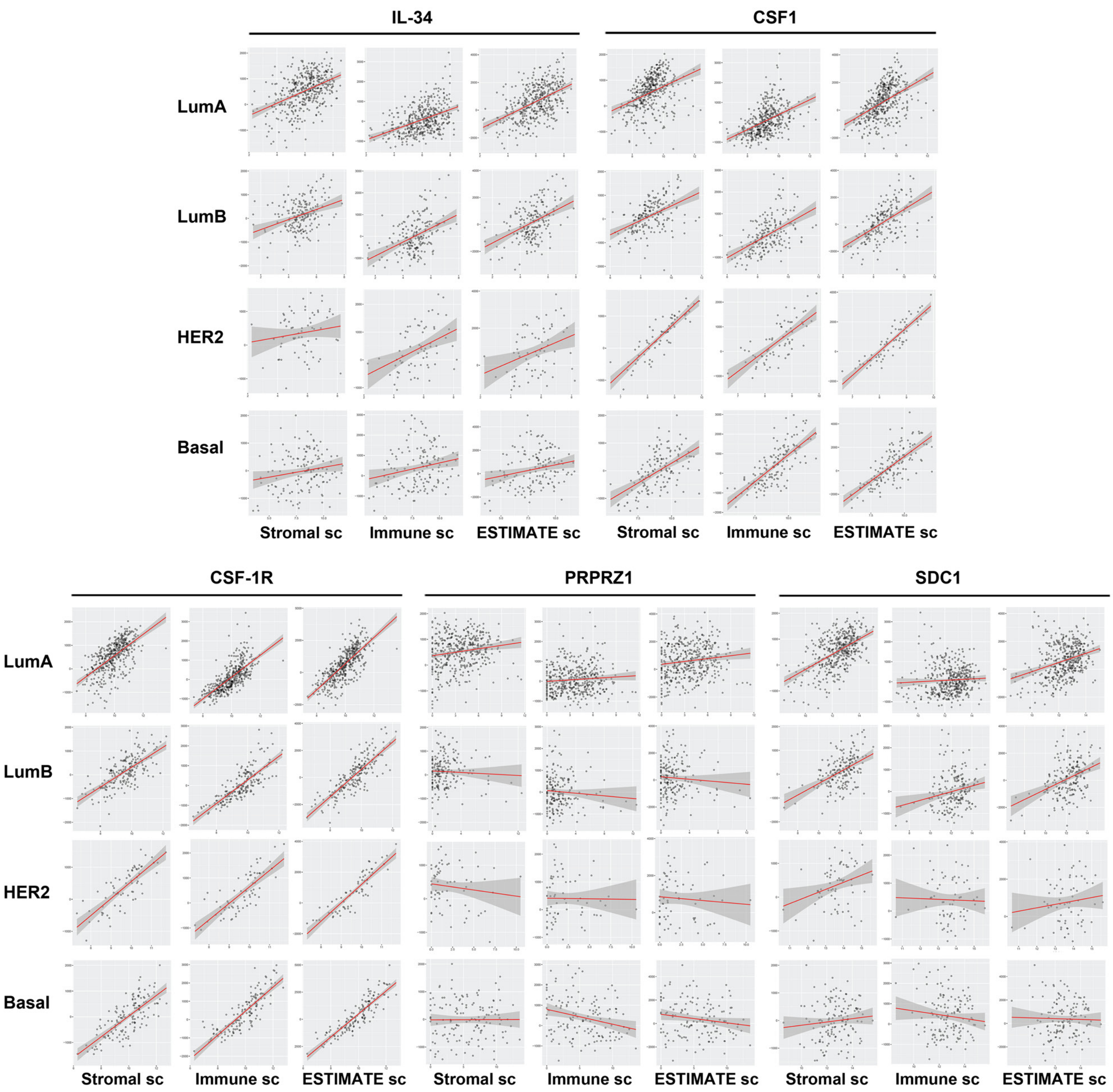

Supplementary Figure 4: Association between mRNA expression of 5 genes and stromal cell/immune cell infiltration in breast tumors stratified by PAM50 calls. Scatter plots demonstrate the correlation between *IL-34*, *CSF-1*, *CSF-1R*, *PTPRZ1* and *SDC1* mRNA expression (log2 scale) and stromal, immune and ESTIMATE scores (obtained from ESTIMATE database) in luminal A (LumA), luminal B (LumB), HER2-enriched and basal-like tumor samples from the TCGA BRCA dataset. Each circle represents a single tumor sample. Regression lines and confidence intervals are shown in red and grey, respectively.

Supplementary Table 1: Immune cell landscape compared with gene expression

	Tumor purity<br>r (*p*)	B cell<br>r (*p*)	CD8+ T cell<br>r (*p*)	CD4+ T cell<br>r (*p*)	Macrophage<br>r (*p*)	Neutrophil<br>r (*p*)	Dendritic cell<br>r (*p*)
IL-34	**−0.445 (<0.0001)**	0.056 (ns)	0.026 (ns)	**0.311 (<0.0001)**	**−0.147 (<0.0001)**	**0.126 (<0.0001)**	**0.195 (<0.0001)**
CSF-1	**−0.379 (<0.0001)**	**0.241 (<0.0001)**	**0.268 (<0.0001)**	**0.400 (<0.0001)**	**0.381 (<0.0001)**	**0.523 (<0.0001)**	**0.479 (<0.0001)**
CSF-1R	**−0.465 (<0.0001)**	**0.414 (<0.0001)**	**0.375 (<0.0001)**	**0.566 (<0.0001)**	**0.549 (<0.0001)**	**0.696 (<0.0001)**	**0.746 (<0.0001)**
PTPRZ1	**−0.237 (<0.0001)**	**−0.123 (<0.001)**	0.017 (ns)	0.047 (ns)	−0.008 (ns)	−0.019 (ns)	−0.001 (ns)
SDC1	**−0.153 (<0.0001)**	0.018 (ns)	−0.057 (ns)	−0.015 (ns)	**0.108 (0.0007)**	0.034 (ns)	**0.107 (<0.001)**

Pearson's product-moment correlation of immune cell landscape of breast cancer compared with TCGA gene expression of *IL-34*, *CSF-1*, *CSF-1R*, *PTPRZ1*, and *SDC1* (TIMER).
r, Pearson's correlation coefficient; *p*, *p*-value; ns, not significant.

Supplementary Table 2: Macrophage infiltration scores and stromal score compared with gene expression

	Monocytes<br>r (*p*)	M0<br>r (*p*)	M1<br>r (*p*)	M2<br>r (*p*)	Stromal sc<br>r (*p*)
IL-34	−0.09 (0.002)	0.04 (ns)	**0.31 (<0.0001)**	**−0.40 (<0.0001)**	**0.20 (<0.0001)**
CSF-1	**0.13 (<0.0001)**	−0.08 (0.012)	**0.17 (<0.0001)**	0.02 (ns)	**0.46 (<0.0001)**
CSF-1R	0.05 (ns)	−0.01 (ns)	**0.20 (<0.0001)**	−0.03 (ns)	**0.64 (<0.0001)**
PTPRZ1	−0.04 (ns)	0.04 (ns)	**0.10 (<0.001)**	**−0.21 (<0.0001)**	**0.09 (0.003)**
SDC1	−0.06 (0.034)	**0.17 (<0.0001)**	0.07 (0.017)	−0.01 (ns)	**0.33 (<0.0001)**

Pearson's product-moment correlation between *IL-34*, *CSF-1*, *CSF-1R*, *PTPRZ1* and *SDC1* mRNA expression and M0, M1, M2 and monocyte infiltration scores (obtained from TCIA database) in tumor samples from the TCGA BRCA dataset. M0, M0 macrophages; M1, M1 macrophages; M2, M2 macrophages; Stromal sc, Stromal score; r, Pearson's correlation coefficient; *p*, *p*-value; ns, not significant.

Supplementary Table 3: Macrophage infiltration and stromal cell/immune cell infiltration in breast tumors stratified by PAM50 calls

Luminal A subtype - Pearson's product-moment correlation

	**M0**	**M1**	**M2**	**Stromal sc**	**Immune sc**	**ESTIMATE sc**
	**r (*p*)**	**r (*p*)**	**r (*p*)**	**r (*p*)**	**r (*p*)**	**r (*p*)**
IL-34	**−0.14 (0.003)**	**0.44 (<0.0001)**	**−0.52 (<0.0001)**	**0.45 (<0.0001)**	**0.44 (<0.0001)**	**0.50 (<0.0001)**
CSF-1	0.01 (ns)	0.03 (ns)	**0.10 (0.0496)**	**0.38 (<0.0001)**	**0.46 (<0.0001)**	**0.47 (<0.0001)**
CSF-1R	0.07 (ns)	**0.12 (0.016)**	−0.02 (ns)	**0.61 (<0.0001)**	**0.71 (<0.0001)**	**0.74 (<0.0001)**
PTPRZ1	**−0.15 (0.003)**	**0.23 (<0.0001)**	**−0.36 (<0.0001)**	**0.17 (<0.001)**	0.08 (ns)	**0.14 (0.005)**
SDC1	**0.23 (<0.0001)**	0.04 (ns)	0.03 (ns)	**0.53 (<0.0001)**	0.06 (ns)	**0.32 (<0.0001)**

M0, M0 macrophages; M1, M1 macrophages; M2, M2 macrophages; Stromal sc, Stromal score; Immune sc, Immune score; Estimate sc, Estimate score; r, Pearson´s correlation coefficient; *p*, *p*-value; ns, not significant.

Luminal B subtype - Pearson's product-moment correlation

	**M0**	**M1**	**M2**	**Stromal sc**	**Immune sc**	**ESTIMATE sc**
	**r (*p*)**	**r (*p*)**	**r (*p*)**	**r (*p*)**	**r (*p*)**	**r (*p*)**
IL-34	0.08 (ns)	**0.32 (<0.0001)**	**−0.40 (<0.0001)**	**0.35 (<0.0001)**	**0.44 (<0.0001)**	**0.44 (<0.0001)**
CSF-1	0.004 (ns)	0.13 (ns)	**0.22 (0.002)**	**0.48 (<0.0001)**	**0.52 (<0.0001)**	**0.56 (<0.0001)**
CSF-1R	−0.002 (ns)	**0.28 (<0.0001)**	0.11 (ns)	**0.67 (<0.0001)**	**0.79 (<0.0001)**	**0.82 (<0.0001)**
PTPRZ1	0.06 (ns)	0.03 (ns)	−0.02 (ns)	−0.05 (ns)	−0.08 (ns)	−0.08 (ns)
SDC1	0.10 (ns)	**0.19 (0.006)**	0.05 (ns)	**0.48 (<0.0001)**	**0.22 (0.002)**	**0.38 (<0.0001)**

M0, M0 macrophages; M1, M1 macrophages; M2, M2 macrophages; Stromal sc, Stromal score; Immune sc, Immune score; Estimate sc, Estimate score; r, Pearson´s correlation coefficient; *p*, *p*-value; ns, not significant.

HER2 subtype - Pearson's product-moment correlation

	**M0**	**M1**	**M2**	**Stromal sc**	**Immune sc**	**ESTIMATE sc**
	**r (*p*)**	**r (*p*)**	**r (*p*)**	**r (*p*)**	**r (*p*)**	**r (*p*)**
IL-34	0.15 (ns)	0.14 (ns)	**−0.30 (0.014)**	0.15 (ns)	**0.42 (<0.001)**	**0.34 (0.005)**
CSF-1	−0.10 (ns)	0.17 (ns)	−0.15 (ns)	**0.87 (<0.0001)**	**0.72 (<0.0001)**	**0.90 (<0.0001)**
CSF-1R	−0.07 (ns)	0.22 (ns)	−0.20 (ns)	**0.79 (<0.0001)**	**0.78 (<0.0001)**	**0.89 (<0.0001)**
PTPRZ1	−0.05 (ns)	0.24 (ns)	−0.19 (ns)	−0.14 (ns)	−0.01 (ns)	−0.08 (ns)
SDC1	**0.33 (0.006)**	**−0.26 (0.032)**	**0.30 (0.012)**	**0.30 (0.013)**	−0.03 (ns)	0.13 (ns)

M0, M0 macrophages; M1, M1 macrophages; M2, M2 macrophages; Stromal sc, Stromal score; Immune sc, Immune score; Estimate sc, Estimate score; r, Pearson´s correlation coefficient; *p*, *p*-value; ns, not significant.

Basal subtype - Pearson's product-moment correlation

	**M0**	**M1**	**M2**	**Stromal sc**	**Immune sc**	**ESTIMATE sc**
	**r (*p*)**	**r (*p*)**	**r (*p*)**	**r (*p*)**	**r (*p*)**	**r (*p*)**
IL-34	0.13 (ns)	0.14 (ns)	−0.16 (ns)	**0.18 (0.031)**	**0.22 (0.010)**	**0.23 (0.008)**
CSF-1	−0.13 (ns)	**0.38 (<0.0001)**	**−0.19 (0.025)**	**0.54 (<0.0001)**	**0.73 (<0.0001)**	**0.72 (<0.0001)**
CSF-1R	−0.05 (ns)	**0.30 (<0.001)**	−0.15 (ns)	**0.73 (<0.0001)**	**0.84 (<0.0001)**	**0.88 (<0.0001)**
PTPRZ1	**0.23 (0.005)**	**−0.21 (0.015)**	0.07 (ns)	0.004 (ns)	**−0.26 (0.002)**	−0.16 (ns)
SDC1	0.07 (ns)	−0.13 (ns)	0.09 (ns)	0.10 (ns)	−0.12 (ns)	−0.03 (ns)

Association between mRNA expression of 5 genes and macrophage infiltration as well as stromal cell/immune cell infiltration in breast tumors stratified by PAM50 calls from the TCGA BRCA dataset.
M0, M0 macrophages; M1, M1 macrophages; M2, M2 macrophages; Stromal sc, Stromal score; Immune sc, Immune score; ESTIMATE sc, Estimate score; r, Pearson´s correlation coefficient; *p*, *p*-value; ns, not significant.
M0, M1, M2 (TCIA database); Stromal sc, Immune sc, ESTIMATE sc (ESTIMATE database).
